# Loss of PTEN Facilitates Rosiglitazone-Mediated Enhancement of Platinum(IV) Complex LA-12-Induced Apoptosis in Colon Cancer Cells

**DOI:** 10.1371/journal.pone.0141020

**Published:** 2015-10-22

**Authors:** Jarmila Lauková, Alois Kozubík, Jiřina Hofmanová, Jana Nekvindová, Petr Sova, Mary Pat Moyer, Jiří Ehrmann, Alena Hyršlová Vaculová

**Affiliations:** 1 Department of Cytokinetics, Institute of Biophysics, Academy of Sciences of the Czech Republic, v.v.i., Brno, Czech Republic; 2 Department of Animal Physiology and Immunology, Institute of Experimental Biology, Faculty of Science, Masaryk University, Brno, Czech Republic; 3 Institute of Clinical Biochemistry and Diagnostics, University Hospital Hradec Kralove, Hradec Kralove, Czech Republic; 4 Platinum Pharmaceuticals, a.s., Brno, Czech Republic; 5 INCELL Corporation LLC, San Antonio, Texas, United States of America; 6 Department of Clinical and Molecular Pathology, Faculty of Medicine and Dentistry, Palacky University Olomouc, Olomouc, Czech Republic; North Carolina State University, UNITED STATES

## Abstract

We demonstrated for the first time an outstanding ability of rosiglitazone to mediate a profound enhancement of LA-12-induced apoptosis associated with activation of mitochondrial pathway in human colon cancer cells. This effect was preferentially observed in the G1 cell cycle phase, independent on p53 and PPARγ proteins, and accompanied with significant changes of selected Bcl-2 family protein levels. Further stimulation of cooperative synergic cytotoxic action of rosiglitazone and LA-12 was demonstrated in the cells deficient for PTEN, where mitochondrial apoptotic pathway was more stimulated and G1-phase-associated dying was reinforced. Our results suggest that combined treatment with rosiglitazone and LA-12 might be promising anticancer strategy in colon-derived tumours regardless of their p53 status, and also favourable in those defective in PTEN function.

## Introduction

Peroxisome proliferator-activated receptor γ (PPARγ) is a member of the nuclear hormone receptor superfamily of ligand-activated transcription factors that are involved in regulation of energy metabolism, cancer development and anti-inflammatory response [1]. Although a main role of PPARγ has been shown in the adipocyte differentiation and insulin sensitisation [2], PPARγ is also well-known to affect growth and cell cycle [3, 4], differentiation [5] and apoptosis [6] of various types of cancer cells including colon. Similarly as in adipocytes, PPARγ expression is also maintained at relatively high levels in numerous human colon cancer cell lines and primary colon tumours [7]. The mutations of PPARγ gene have been reported as rare event in human malignancies including colon [8]. It has been suggested that PPARγ-induced gene regulation might contribute to tumorigenesis, but the significance of this receptor pathway in colon cancer development and treatment still remains controversial.

Rosiglitazone, a synthetic ligand of PPARγ is a widely used anti-diabetic agent from the family of drugs called thiazolidinediones. Due to its ability to inhibit proliferation and/or induce cancer cell death, rosiglitazone has also been examined in numerous studies focused on cancer treatment. Although an insufficient antitumor effectiveness of rosiglitazone has been shown in many cases when used in monotherapy, its promising potential as an adjuvant combined with radiation [9] or various types of antineoplastic agents has been reported. Rosiglitazone enhanced the colon cancer cell sensitivity to the cytotoxic effects of 5-FU [10], cytokine TRAIL [11] or all-trans retinoic acid [12]. Interestingly, additive/synergicanticancer effects of rosiglitazone and conventionally used platinum-based drugs cisplatin or carboplatin have been demonstrated in colon, lung or ovarian cancer cell lines *in vitro* [13, 14]. Combination of carboplatin and rosiglitazone reduced the incidence of polyp formation in mice model of azoxymethane-induced colon carcinogenesis [13], the tumour size in nude mice with subcutaneously injected A549 lung cancer cell-derived xenografts [13] or induced a regression of K-Ras-driven murine lung adenocarcinomas [14]. Pretreatment with rosiglitazone also synergized anticancer activity of cisplatin in DMBA-induced mammary tumours in rats [15]. Although some molecular mechanisms behind these effects have been suggested, many of them still remain to be clarified. Moreover, a complete lack of the information exists regarding the potential cooperative anticancer effects of rosiglitazone with novel platinum-based chemotherapeutic drugs.

LA-12, (OC-6-43)-bis(acetato)(1-adamantylamine)amminedichloroplatinum(IV), represents a recently introduced platinum(IV) complex containing a bulky hydrophobic ligand 1-adamantylamine, enabling its higher hydrophobicity compared to other platinum-based drugs such as cisplatin [16]. The action of LA-12 has been intensively studied by us and others both *in vitro* and *in vivo*, and the results highlighted its favourable anticancer potential over several conventionally used platinum-based chemotherapeutic drugs [17]. LA-12 exerted a strong cytotoxic effect in various cisplatin-resistant cancer cell lines of different origin including colon [18–20]. In addition, LA-12 could overcome confluence-dependent resistance of colon cancer cells that was described in the platinum(II) compounds cisplatin and oxaliplatin [21]. We further demonstrated that a higher efficacy of LA-12 in colon cancer cells was associated with its ability to overcome the block in M-phase entry triggered by oxaliplatin in order to repair the cellular damage [22]. Recently, we reported on a higher/unique ability of LA-12 compared to cisplatin to induce upregulation of several important proteins involved in apoptosis regulation such as Noxa, Bim, c-Myc or DR5 [23, 24]. Furthermore, the cellular uptake of LA-12 has been shown faster and more effective compared to cisplatin in human non-small cell lung carcinoma [25]. Promising anticancer properties of LA-12 were also demonstrated *in vivo* in nude mice bearing human carcinoma xenografts of colon, prostate and ovarian origin, where LA-12 was more effective in tumour elimination compared to satraplatin [26]. However, neither the detailed molecular mechanisms involved in the cytotoxic and cytostatic action of LA-12 in colon cancer cells are still fully understood, nor are its potential applications in combined therapy.

In present study, we were the first to demonstrate the ability of rosiglitazone to stimulate antiproliferative and apoptotic response triggered by LA-12 in HCT116 human colon adenocarcinoma cells. We investigated the molecular mechanisms responsible for the cooperative action of the drugs, with a particular focus on the modulation of the cell cycle progression, PTEN involvement and activation of mitochondrial apoptotic pathway. The cytotoxic response elicited by the combination of rosiglitazone and LA-12 was also investigated in other colon cancer cells lines and the cells derived from normal colon epithelium.

## Materials and Methods

### Cell Culture and treatments

Human colon adenocarcinoma cell lines HCT116 wt (p53^+/+^, Bax^+/-^, Chk2^+/+^, PTEN^+/+^), p53^-/-^, Bax^-/-^, Chk2^-/-^ and PTEN^-/-^ (obtained from Prof. Bert Vogelstein, John Hopkins University, Baltimore, MD, USA, and T. Waldman, Georgetown University School of Medicine, Washington, USA, in 2007) [27] [28] were maintained in McCoy´s 5A medium (Gibco, Invitrogen, Life Technologies, USA), supplemented with penicillin (100 U/ml), streptomycin (0.1 mg/ml) and 10% heat-inactivated foetal bovine serum (FBS, PAA Laboratories GmbH, Pasching, Austria and Gibco, Invitrogen). Human colon adenocarcinoma cells DLD-1 (ATCC, CCL-221, obtained in 2009) and RKO (ATCC, CRL-2577, obtained in 2007) were maintained in RPMI or DMEM (both Gibco, Invitrogen, Life Technologies), respectively, supplemented with penicillin (100 U/ml), streptomycin (0.1 mg/ml) and 10% FBS. The NCM460 human adult normal colon epithelium-derived cell line was received (in 2010) by a Material Transfer Agreement with INCELL Corporation (San Antonio, Texas, USA) [29], and routinely propagated under standard conditions in M3:10TM medium (INCELL Corporation). The cells were cultivated in TPP (TPP Techno Plastic Products AG, Trasadingen, Switzerland) cultivation dishes, flasks or plates in a humidified incubator at 37°C in atmosphere of 5% CO_2_, passaged twice a week after exposure to EDTA/PBS and trypsin solutions. Numbers of cells were determined using a CASY Model TT–Cell Counter and Analyzer (Roche Diagnostics GmbH, Germany).

Twenty-four hours after seeding, the cells were pretreated (24 h) with 50 μM rosiglitazone (RGZ) (5-[[4-[2-(Methyl-2-pyridinylamino)ethoxy]phenyl]methyl]-2,4-thiazolidinedione, Cayman Chemical, Michigan, USA) and subsequently treated (48 h) with 0.75 μM LA-12 ([(OC-6-43)-bis(acetato)(1-adamantylamine)aminedichloroplatinum(IV)], Platinum Pharmaceuticals, a.s., Brno, Czech Republic). MEK1/2 Inhibitor U0126 (#9903, Cell Signaling Technology, Danvers, MA, USA) and pan-caspase inhibitor z-VAD-fmk (#550377, BD Bioscience Pharmingen, San Diego, CA, USA) were added to cells 1 h before treatment with RGZ. Stock solutions were diluted in dimethylsulfoxide (DMSO, Sigma–Aldrich, Prague, Czech Republic).

### Cell Transfection and RNA Interference

RNA transfections were performed using X-tremeGENE siRNA Transfection Reagent (Roche, Basel, Switzerland) or Lipofectamine™ 2000 Transfection Reagent (Invitrogen, Carlsbad, CA, USA) according to the manufacturer’s recommendations. Cells were seeded into McCoy’s medium with 10% FBS, without antibiotics, and incubated overnight. Shortly before transfection medium was changed for Opti-MEM® I Reduced Serum Medium (Gibco, Invitrogen). The cells were transfected with 100 nM siRNA duplexes directed against PPARγ(#29455, Santa Cruz Biotechnology, Santa Cruz, CA, USA) or non-target control siRNA (#37007, Santa Cruz Biotechnology). After 4 h, medium was changed for McCoy’s medium with 10% FBS (PAA Laboratories GmbH and Gibco, Invitrogen) with antibiotics. Twenty-four hours after transfection, the cells were pretreated (24 h) with RGZ and subsequently treated (48 h) with LA-12.

### Immunoblotting analysis

The cells were lysed in 1% SDS lysis buffer containing protease inhibitor cocktail (#P2714, Sigma–Aldrich) or Protease Inhibitor Cocktail Set I (#5391313, Calbiochem, Merck Millipore, Bedford, MA, USA), for phosphoprotein detection NaVO_4_ and NaF were added to the lysis solution. DC™ Protein Assay (#500–0113, Bio-Rad Laboratories, Prague, Czech Republic) was used for protein quantification. Proteins were then separated on 15% SDS-polyacrylamide gel, and blotted onto a PVDF membrane (Merck Millipore, Bedford, MA, USA). The membranes were blocked in 5% non-fat milk or BSA for 1 h at RT, washed with TBS (50 mM Tris–HCl, 150 mM NaCl and 0.1% Tween), and incubated overnight in primary antibodies diluted in 5% non-fat milk at 4°C. Immunodetection was carried out using the following primary antibodies: monoclonal mouse anti-p53 (1:000, DO-1, #126) raised against amino acids 11–25 of p53 of human origin, polyclonal rabbit anti-Akt (1:500, #8312) against amino acids 345–480 of Akt1 of human origin, monoclonal mouse anti-cyclin B1 (1:1000, #245) against a recombinant protein corresponding to human cyclin B1, monoclonal mouse anti-cyclin D1 (1:1000, #20044) against recombinant full length human protein, monoclonal mouse anti-PTEN (1:500, #7974) against amino acids 388–400 of PTEN of human origin, polyclonal rabbit anti-p21 (1:000, #397) raised against a peptide mapping at the C-terminus of p21 of human origin, polyclonal rabbit anti-p27 (1:1000, #528) raised against a peptide mapping at the C-terminus of p27 of human origin (all from Santa Cruz Biotechnology), polyclonal rabbit anti-phospho-Akt (Ser473) (1:500, #9271) produced by immunizing animals with a synthetic phospho-peptide corresponding to residues surrounding Ser473 of mouse Akt–purified by protein A, polyclonal rabbit anti-Bak (1:1000, #3792) produced by immunizing rabbits with a synthetic peptide (KLH-coupled) corresponding to the amino-terminal residues of human Bak–purified by protein A, polyclonal rabbit anti-Bax (1:1000, #2772) produced by immunizing animals with a synthetic peptide (KLH-coupled) corresponding to the amino-terminal residues of human Bax—purified by protein A, polyclonal rabbit anti-Bid (1:1000, #2002) produced by immunizing animals with a synthetic peptide (KLH-coupled) corresponding to residues surrounding the cleavage site of human Bid–purified by protein A, monoclonal rabbit anti-Bim (1:1000, #2933) produced by immunizing animals with a synthetic peptide corresponding to residues surrounding Pro25 of Bim, monoclonal mouse anti-Chk2 (1:000, #3440) produced by immunizing animals with truncated recombinant GST-Chk2, polyclonal rabbit anti-cleaved caspase-3 (1:500, #9661) produced by immunizing animals with a synthetic peptide corresponding to amino-terminal residues adjacent to (Asp175) in human caspase-3, polyclonal rabbit anti-cleaved caspase-9 (1:500, #9505) produced by immunizing animals with a synthetic peptide corresponding to amino terminus residues surrounding to Asp330 of human caspase-9 –purified by protein A, polyclonal rabbit anti-ERK (1:1000, #9102) produced by immunizing animals with a synthetic peptide (KLH-coupled) derived from a sequence in the C-terminus of rat p44 MAP Kinase–purified by protein A, polyclonal rabbit anti-phospho-ERK1/2 (Thr202/Tyr204) (1:1000, #9101) produced by immunizing animals with a synthetic phospho-peptide corresponding to residues surrounding Thr202/Tyr204 of human p44 MAP kinase–purified by protein A, polyclonal rabbit anti-cleaved PARP (Asp214) (1:1000, #9541) produced by immunizing animals with a synthetic peptide corresponding to carboxy-terminus residues surrounding Asp214 in human PARP–purified by protein A, polyclonal rabbit anti-peroxisome proliferator-activated receptor γ(PPARγ) (1:500, #2435) produced by immunizing rabbits with a synthetic peptide corresponding to residues surrounding Asp69 of human PPARγ, polyclonal rabbit anti-Puma (1:1000, #4976), produced by immunizing animals with a synthetic peptide corresponding to the carboxy-terminal region of human Puma–purified by protein A, monoclonal rabbit anti-survivin (1:1000, #2808) produced by immunizing animals with a synthetic peptide corresponding to residues surrounding cysteine 60 of human survivin (all from Cell Signaling Technology), mouse monoclonal anti-Noxa (OP180; Calbiochem-MERC, Prague, Czech Republic) with immunogen of GST-fusion with human recombinant Noxa, mouse anti-cytochrome c (1:500, #556433, BD Pharmingen™, San Jose, USA) with synthetic peptides of pigeon cytochrome c as immunogen, monoclonal mouse anti-complex IV subunit II (anti-cyt oxidase subunit II) (#A-6404, Invitrogen™, Life Technologies) with human complex IV subunit II as immunogen. The membranes were then washed and incubated with secondary antibodies—anti-mouse IgG (1:3000, #NA931) and anti-rabbit IgG antibody (1:3000, #NA934) (both from Amersham Biosciences, Piscataway, NJ, USA) for 1 h at RT. Detection of the antibody complexes was performed with horseradish peroxidase substrates Immobilon Western Chemiluminescent HRP Substrate (#WBKLS0500, Merck Millipore, Bedford, MA, USA). Anti-β-actin antibody (1:5000, A5441; Sigma-Aldrich), with slightly modified β-cytoplasmic actin N-terminal peptide, Ac-Asp-Asp-Asp-Ile-Ala-Ala-Leu-Val-Ile-Asp-Asn-Gly-Ser-Gly-Lys, conjugated to KLH as immunogen, was used for a loading control.

### Preparation of cell fractions

The cells were harvested by trypsinization, centrifuged at 200 g for 5 min, resuspended in fraction buffer (150 mM KCl, 1mM MgCl2, 0.2 mM EGTA, 5 mM Tris and 0.01% digitonin pH 7.2–7.4) and left in RT for 20 min to lyse. Lysed cells were centrifuged at 13 000 rpm, supernatants were used for cytoplasmic fractions and pellets were resuspended in the same amount of fraction buffer mixed with 4x Laemmlli buffer, boiled for 10 min and the samples after protein quantification were used in immunoblotting analysis.

### Analysis of mitochondrial membrane potential (MMP)

Detection of MMP was performed using 0.2 μM tetramethylrhodamine ethyl ester perchlorate (TMRE; Molecular Probes®, Eugene, OR, USA) as described previously [30] and analysed using flow cytometry (FACS Calibur^TM^, Becton Dickinson, San Jose, CA, USA). The data were evaluated by CellQuest software (Becton Dickinson) and the results were expressed as the percentages of the cells with decreased MMP.

### Annexin V/ propidium iodide apoptosis assay

For externalization of phosphatidyl serine as a marker of apoptosis, the cells were harvested, washed with PBS and stained with annexin V FITC-conjugated antibody (#ANXV-FT100, Apronex, Prague, Czech Republic) for 20 min in RT with manufactures’ specific supplied buffer. Just before analysis, 5 μg/ml propidium iodide (PI) (#P-4170, Sigma-Aldrich) was added. Fluorescence was then measured using flow cytometer (FACS Calibur^TM^, Becton Dickinson). Using Cell Quest software, the results were expressed as the percentage of the cells positive for annexin V and negative for propidium iodide (apoptotic).

### Cell cycle analysis

The cells were processed as described previously [22] and analysed by flow cytometry (FACSVerse^TM^; Becton Dickinson); minimum of 20 000 events were collected per sample. Data were analysed using ModFit LT version 3.1 software (Verity Software House, Topsham, ME, USA). Debris and doublet cells were excluded and only single cells were taken for cell cycle analysis. Results were expressed as the percentage of the cells in G1, S and G2/M phase.

### Active caspase-3 detection

The cells were harvested, washed in PBS, fixed and stained using FITC active caspase-3 apoptosis kit (#550480, BD Pharmingen™) according to the manufacturer´s protocol. The percentage of cells with active caspase-3 was detected by flow cytometry (FACS Verse^TM^, Becton Dickinson) and analysed by BD FACSuite software version 1.0.5 (Becton Dickinson).

### Simultaneous detection of active caspase-3 and the cell cycle progression

The cells were harvested, washed in PBS, fixed and stained using FITC active caspase-3 apoptosis kit (#550480, BD Pharmingen™) according to the manufacturer´s protocol. Subsequently, the cells were washed and stained with propidium iodide for cell cycle analysis as described above. The percentage of the cells in G1, S and G2/M phase with active caspase-3 was detected by flow cytometry (FACS Verse^TM^, Becton Dickinson) and analysed by BD FACSuite software version 1.0.5 (Becton Dickinson).

### RNA isolation and real-time RT-PCR

#### RNA isolation

Immediately after collection, 1x10^6^ cells were resuspended in 200 μl PBS, homogenized in 400 μl kit Lysis/Binding Buffer and frozen at -80°C until the time of RNA isolation. Total RNA isolation was performed using High Pure RNA Isolation Kit (Roche, Switzerland) according to manufacturer’s instructions. RNA quantity and purity were assessed by UV-spectroscopy and an aliquot of RNA was reversely transcribed to cDNA.

#### cDNA synthesis

cDNA was synthesized from 1 μg of total RNA by Transcriptor First Strand cDNA Synthesis kit (Roche, Switzerland) according to manufacturer’s instructions using provided oligo-dT primers (1 μl) and random hexamer primers (2 μl) in a total volume of 20 μl.

#### Real-Time quantitative PCR

0.5 μl of each cDNA was analyzed by quantitative PCR using TaqMan Gene Expression Master Mix and TaqMan Gene Expression assays (Life Technologies, USA) CCND1, cyclin D1 (Hs00765553_m1), BIRC5, survivin (Hs04194392_s1), CDKN1A, p21 (Hs00355782_m1), CDKN1B, p27 (Hs01597588_m1), CCNB1, cyclin B1(Hs01030099_m1), HPRT1 (Hs99999909_m1) according to manufacturer’s instructions in a total reaction volume of 10 μl. All PCR reactions were performed in three technical replicates on RotorGene 6000 instrument (Corbett Life Science, Australia). PCR thermal profile was as follows: 50°C for 2 min (UDG incubation), 95°C for 10 min (enzyme activation), followed by 40 cycles of 95°C for 15 s and 60°C for 1 min. NTC and RT- controls were included in each run. Data analysis was performed using the ddCt method with HPRT1 used as the housekeeping gene.

### Statistical data analysis

Data are expressed as Mean +/- S.E.M. of three independent experiments, and statistically analysed by one-way ANOVA followed by Fisher's Least Significant Difference (LSD) test with statistical significance p<0.05, using Statistica for Windows software, V 10 (StatSoft, Inc., Tulsa, OK, USA).

In order to examine in more detail the interactive (synergism or additivity) cytotoxic effects between the drugs, the results of apoptosis analysis (annexin V/propidium iodide assay, flow cytometry, percentage of annexin V positive, propidium iodide negative HCT116 cells) following treatment with several different doses of rosiglitazone (up to 75 μM) and LA-12 (up to 1.5 μM) or their combinations were examined. Construction of regression curves and calculating of the equieffective quantities of agents in the mixture were done using isobolographic analysis in GraphPad Prism 6 software. The drug doses sufficient for synergistic effect were calculated and the type of the response was determined.

## Results

### RGZ enhanced HCT116 human colon cancer cell sensitivity to LA-12-induced caspase-dependent apoptosis

**Fig 1:**
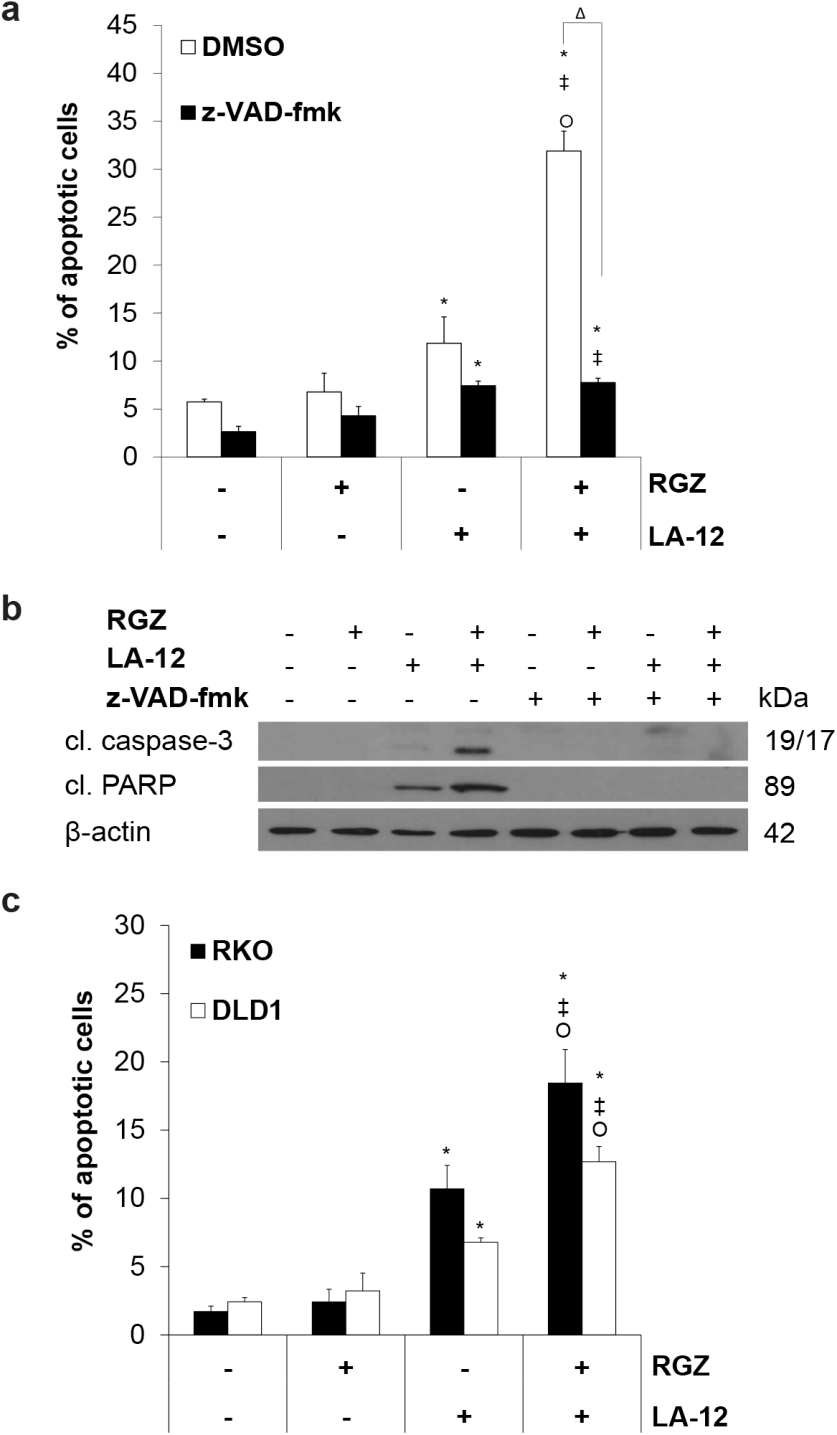
Rosiglitazone-mediated sensitization of HCT116 cells to LA-12-induced apoptosis. (a) Percentage of apoptotic (annexin V positive/propidium iodide negative, flow cytometry) HCT116 wt cells and (b) cleavage of caspase-3 and PARP (Western blotting) following pretreatment (24 h) with rosiglitazone (RGZ, 50 μM) and subsequent treatment (48 h) with LA-12 (0.75 μM), in the absence (DMSO) or presence of z-VAD-fmk (10 μM). (c) Percentage of apoptotic (annexin V positive/propidium iodide negative, flow cytometry) RKO or DLD1 cells following pretreatment (24 h) with rosiglitazone (RGZ, 50 μM) and subsequent treatment (48 h) with LA-12 (0.75 μM). Results are means + S.E.M. or representatives of three independent experiments. Statistical significance: P < 0.05, * versus control, ‡ versus RGZ, Ο versus LA-12, and Δ for with/without z-VAD-fmk.

Pretreatment of HCT116 wt human adenocarcinoma cell line with rosiglitazone effectively stimulated LA-12-induced apoptosis, as demonstrated by significant increase in percentage of the cells with specific phosphatidyl serine externalization (Fig 1A; S1 Fig), and cleavage of caspase-3 and its substrate PARP (Fig 1B). An isobolographic analysis of the apoptotic response of the HCT116 cells to several different doses of rosiglitazone and LA-12 used in combination (see Materials and methods) clearly demonstrated a significant synergic effect (data not shown). Using a pan-caspase inhibitor z-VAD-fmk, we confirmed that the apoptotic effects of the drug combination fully depended on caspase activation (Fig 1A and 1B). The significant stimulation of the apoptotic effects (phosphatidyl serine externalization) induced by LA-12 and rosiglitazone combination compared to the individual drugs was also demonstrated in human colon adenocarconima DLD-1 and RKO cell lines (Fig 1C).

### Mitochondria play an indispensable role in enhancement of HCT116 cell apoptosis following their combined treatment with rosiglitazone and LA-12

**Fig 2:**
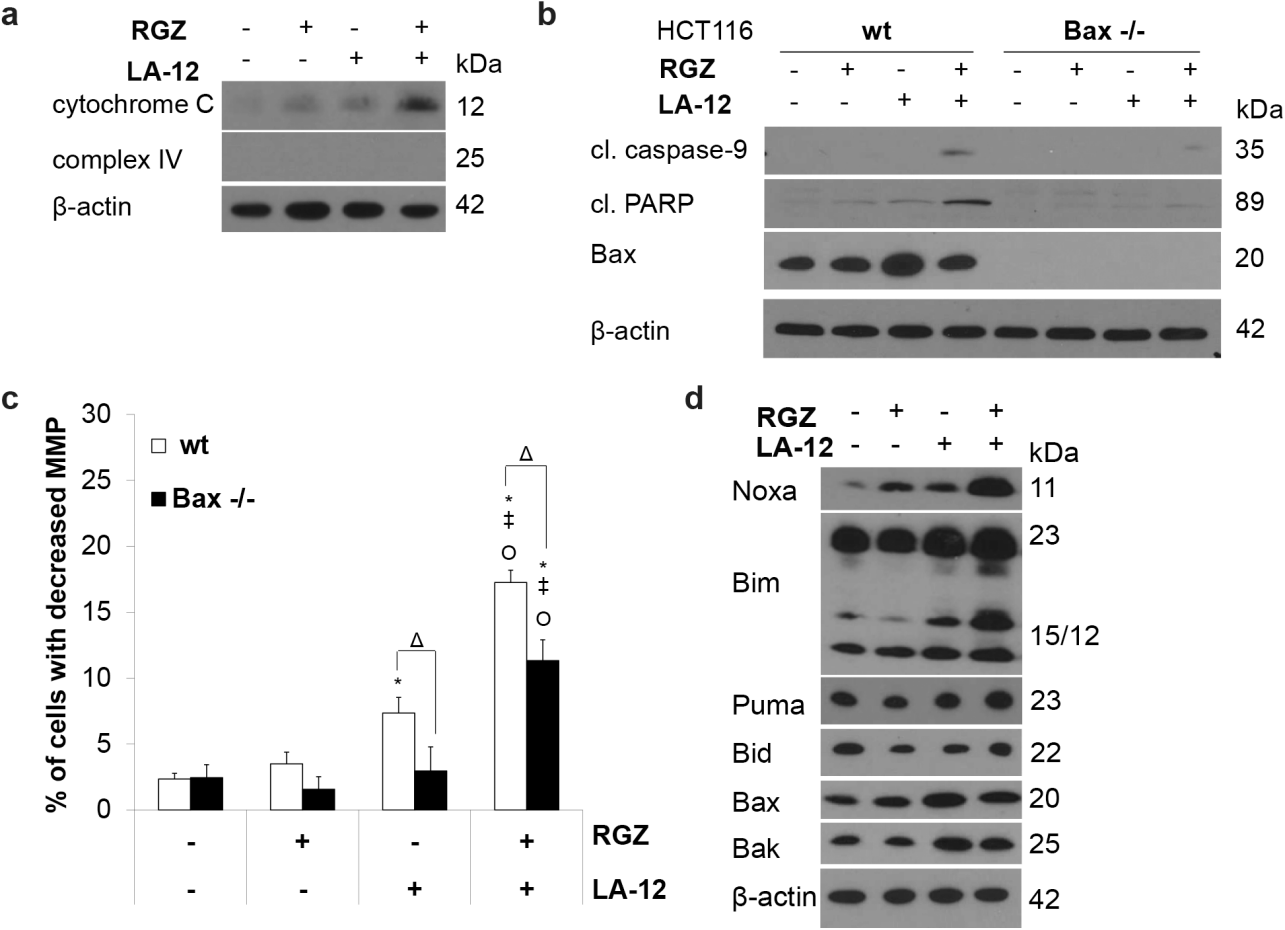
Involvement of mitochondria in cooperative cytotoxic action of rosiglitazone and LA-12. (a) The release of cytochrome c into the cytoplasm (cytoplasmic fraction) of HCT116 cells pretreated (24 h) with rosiglitazone (RGZ, 50 μM) and subsequently treated (48 h) with LA-12 (0.75 μM), detected by Western blotting after cell fractionation. (b) Cleavage of caspase-9, PARP, Bax protein level (Western blotting), and (c) changes in mitochondrial membrane potential (MMP, flow cytometry) in HCT116 wt and Bax-/- cells treated as in a). (d) The level of Noxa, Bim, Puma, Bid, Bax and Bak protein (Western blotting) in HCT116 wt cells treated as in a). Results are means + S.E.M. or representatives of three independent experiments. Statistical significance: P < 0.05, * versus control, ‡ versus RGZ or Ο versus LA-12, and Δ for wt versus Bax-/- cells.

The apoptotic response of HCT116 wt cells treated with the combination of rosiglitazone and LA-12 was associated with stimulation of the mitochondrial pathway, as indicated by the enhanced release of cytochrome c into the cytoplasm (Fig 2A), cleavage of caspase-9 (Fig 2B) and drop of MMP (Fig 2C). A functional role of mitochondria was further confirmed using HCT116 Bax-/- cells, where the apoptotic effects of the drug combination were significantly suppressed. This was demonstrated by a blockage of caspase-9 and PARP cleavage (Fig 2B), and drop of MMP (Fig 2C) in HCT116 Bax-/- cells treated by the combination of rosiglitazone and LA-12 compared to their wt counterparts. Following the HCT116 wt cell treatment with the drug combination, an increased level of pro-apoptotic BH3-only proteins Noxa and Bim (23 and 15 kDa, which corresponds to EL and L form, respectively) was observed, while the level of Puma and Bid remained largely unaffected. A slight increase in Bax and Bak protein level was also apparent in LA-12-treated HCT116 wt cells (Fig 2D).

### The rosiglitazone- and LA-12-induced apoptosis occurs preferentially in G1 phase of HCT116 cell cycle

**Fig 3:**
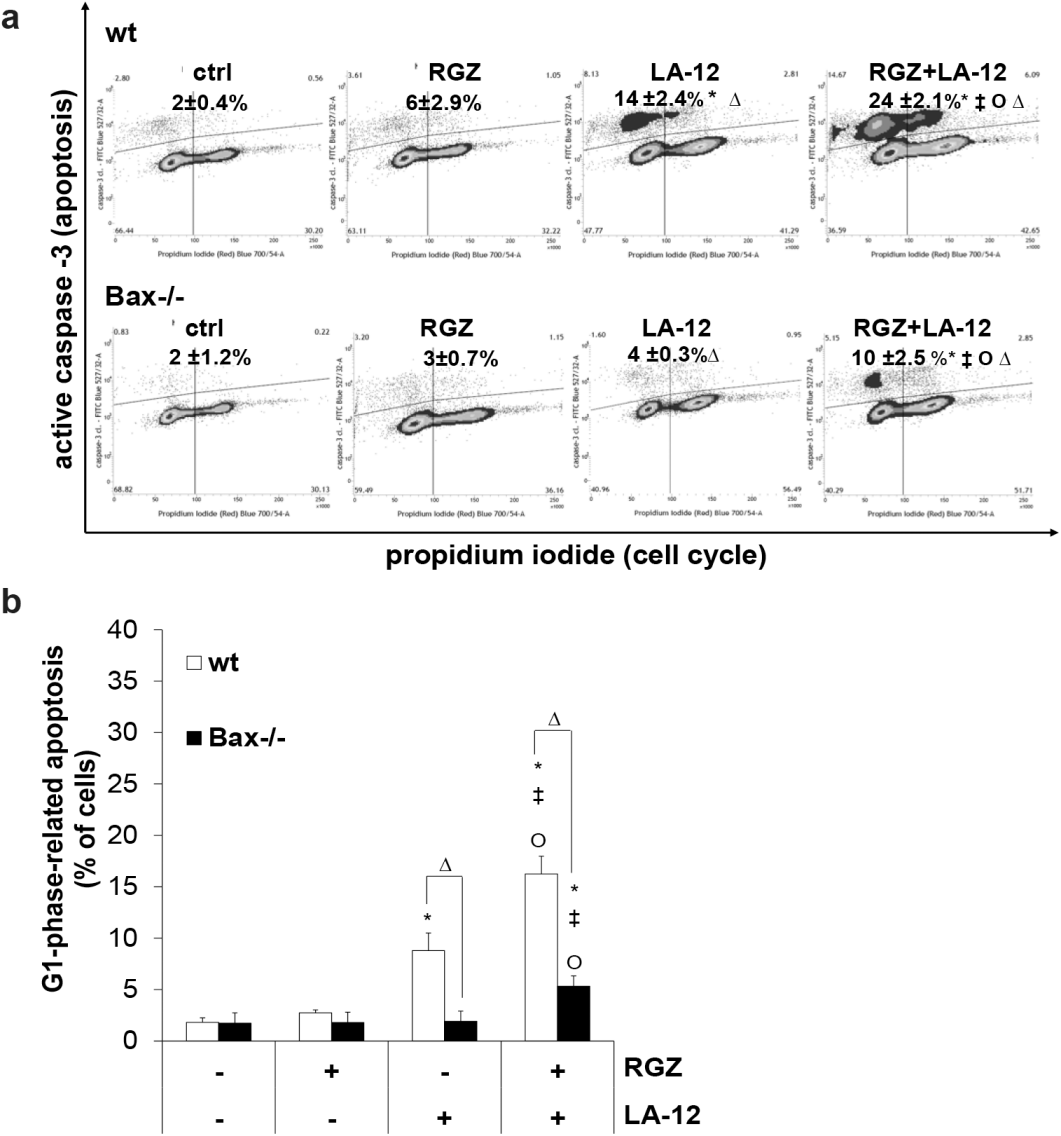
Relationship between HCT116 cell cycle progression and apoptosis induced by combination of rosiglitazone and LA-12. (a) Caspase-3 activation (% of all cells with active caspase-3) related to the cell cycle progression (flow cytometry) in HCT116 wt and Bax-/- cells following their pretreatment (24 h) with rosiglitazone (RGZ, 50 μM), and subsequent treatment (48 h) with LA-12 (0.75 μM) (b) Percentage of apoptotic cells (with active caspase-3) in G1 cell cycle phase, quantification of data from a). Results are means + S.E.M. or representatives of three independent experiments. Statistical significance: P < 0.05, * versus control, ‡ versus RGZ, Ο versus LA-12, and Δ for wt versus Bax-/- cells.

A simultaneous flow cytometry analysis of the cell apoptosis (caspase-3 cleavage) and cell cycle revealed that the cells treated with the drug combination preferentially die from the G1 (and to low extent also S) phase (Fig 3A and 3B). Under the conditions of Bax deficiency, significantly lower percentage of the cells treated with the drug combination underwent apoptosis compared to their Bax expressing counterparts, and these apoptotic cells were in the G1 cell cycle phase (Fig 3A).

**Fig 4:**
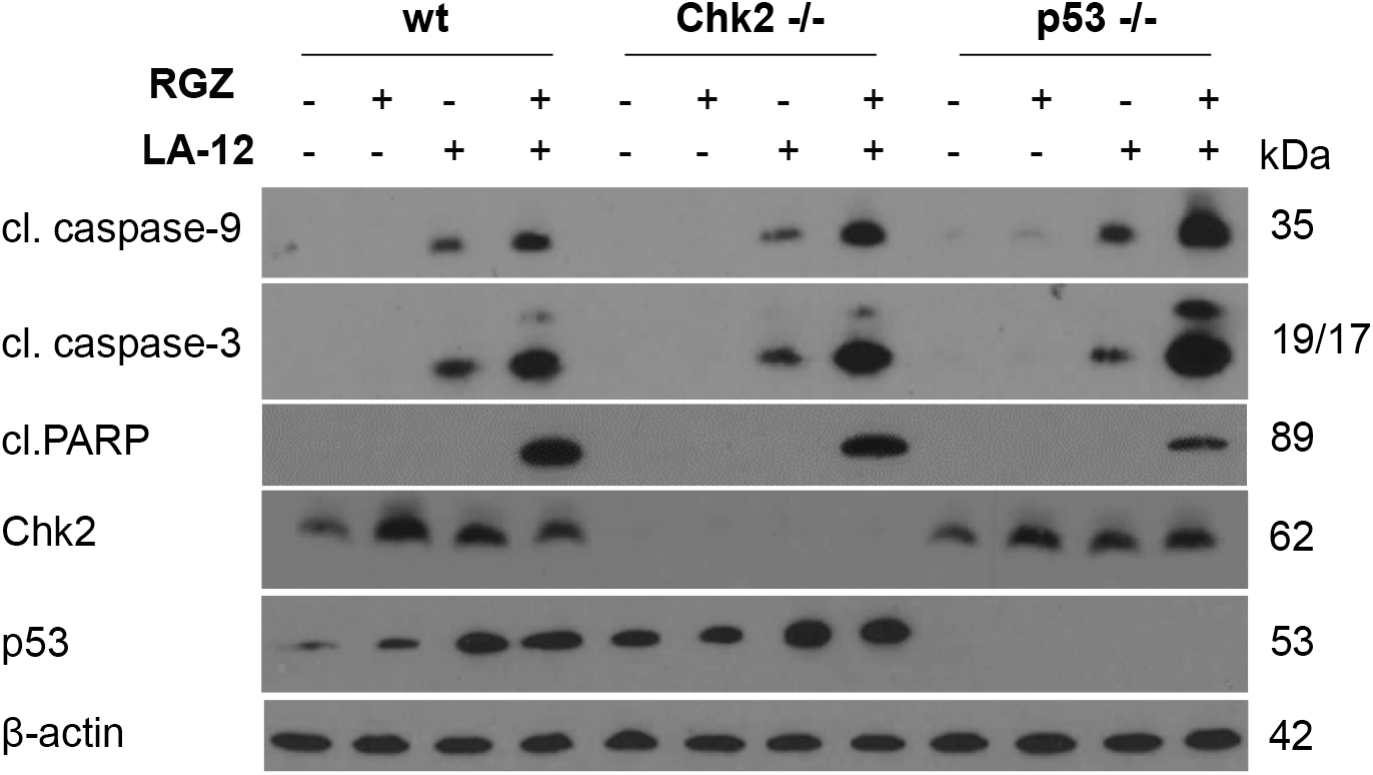
The role of p53 and Chk2 proteins in rosiglitazone-mediated enhancement of LA-12-induced apoptosis. Cleavage of caspase-9, caspase-3, PARP and the level of Chk2 and p53 proteins in HCT116 wt, Chk2-/- and p53-/- cells pretreated (24 h) with rosiglitazone (RGZ, 50 μM), and subsequently treated (48 h) with LA-12 (0.75 μM), detected by Western blotting. Results are representatives of three independent experiments.

As p53 and Chk2 may act as important cell cycle (including G1- or M-phase entry) and apoptosis regulators, we investigated their possible involvement in modulation of rosiglitazone-mediated enhancement of HCT116 cell sensitivity to LA-12-induced apoptosis. Our results showed that both p53 and Chk2 are dispensable for the apoptosis triggered by the drug combination, as similar response (caspase-3, caspase-9, PARP cleavage) was observed in parental and appropriate knock out (p53-/-, Chk2-/-) cell lines (Fig 4).

### PTEN deficiency supported G1 phase-related enhancement of mitochondria-dependent apoptosis induced by rosiglitazone and LA-12 in HCT116 cells

**Fig 5:**
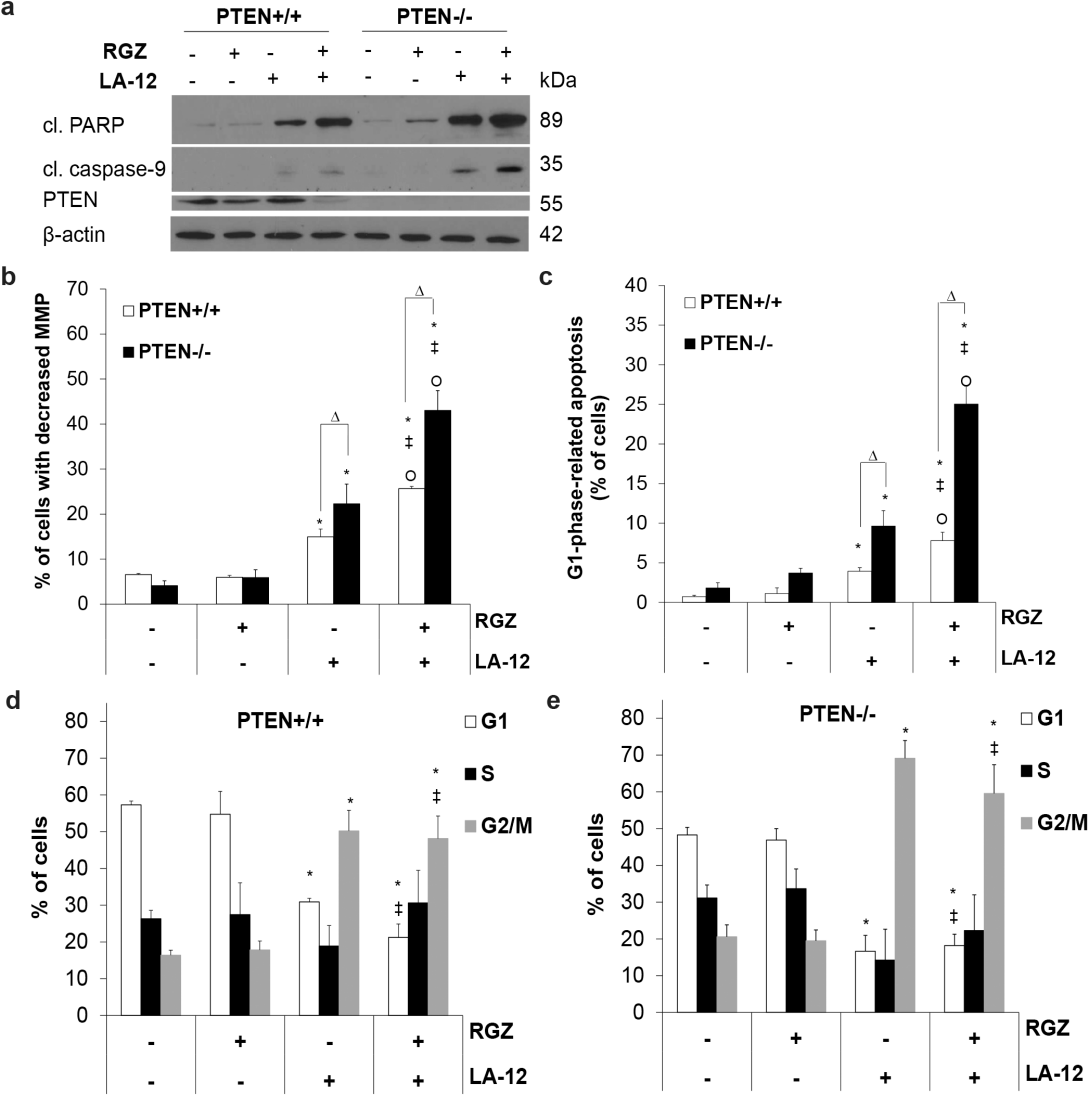
A functional importance of PTEN in rosiglitazone-mediated sensitization of HCT116 cells to LA-12-induced apoptosis. (a) Cleavage of PARP, caspase-9 and PTEN level (Western blotting), (b) changes in mitochondrial membrane potential (MMP, flow cytometry), and (c) percentage of apoptotic cells (with active caspase-3) in G1 cell cycle phase in HCT116 wt or PTEN-/- cells pretreated (24 h) with rosiglitazone (RGZ, 50 μM), and subsequently treated (48 h) with LA-12 (0.75 μM). (d, e) Percentage of HCT116 wt and PTEN-/- cells in individual cell cycle phases (flow cytometry) following the treatments specified in a-c). Results are means + S.E.M. or representatives of three independent experiments. Statistical significance: P < 0.05, * versus control, ‡ versus RGZ, Ο versus LA-12, and Δ for wt versus PTEN-/- cells.

Combined treatment of HCT116 wt cells with rosiglitazone and LA-12 induced a decrease in PTEN level (Fig 5A), which was accompanied by an increased activation of Akt (phosphorylation at Ser473), without changes in the total protein kinase level (S2 Fig). In order to investigate the functional role of PTEN, the cytotoxic response to the drug combination was compared in HCT116 cells expressing or lacking the protein. HCT116 PTEN-/- cells were more sensitive to the apoptotic effects of LA-12 alone and even more to its combination with rosiglitazone, as showed by an enhanced PARP and caspase-9 cleavage (Fig 5A), and an increased drop of mitochondrial membrane potential (Fig 5B) compared to their HCT116 wt counterparts. A preferential killing from the G1 cell cycle phase induced by the drug combination in the HCT116 wt cells was further even more supported in the absence of PTEN, where the percentage of apoptotic cells (with caspase-3 activation) increased significantly (Fig 5C). The stimulation of drug combination-induced G1-phase-related apoptosis was also apparent in later time points of the treatments (data not shown).

While rosiglitazone alone did not affect the cell cycle distribution compared to control, we observed a significant decrease in percentage of HCT116 wt cells in G1 phase, and simultaneous increase in G2/M phase after their exposure to LA-12 or its combination with rosiglitazone (Fig 5D). Compared to the wt counterparts, a further remarkable increase in percentage of cells in G2 phase was apparent in PTEN-/- cells treated with LA-12 or its combination with rosiglitazone (Fig 5E). Simultaneously, we detected a remarkable decrease in the level of several cell cycle regulatory proteins, namely cyclin D1, B1, p21, p27 and survivin in HCT116 wt cells treated with the drug combination compared to LA-12 alone (S3 Fig). In a good accordance, the apparent decrease of the amount of these molecules in the cells treated with the drug combination versus LA-12 alone was also detected at the level of mRNA (S4 Fig). In addition, a further more intensive drop in cyclin B1, p21, p27 and survivin protein level was apparent in PTEN-/- cells treated with the combination of rosiglitazone and LA-12 compared to the similarly treated wt counterparts, while the opposite trends were evident for cyclin D1 (S3 Fig). The described differences at the protein level between the PTEN +/+ and -/- cells incubated in the presence of the drug combination were further well correlated at the level of mRNA for p21 and cyclin D1, important regulators of G1 phase progression, but not evident in case of p27, cyclin B1 and survivin (S4 Fig).

In addition to Akt, we detected a significant increase in ERK1/2 phosphorylation in HCT116 wt cells treated with the combination of rosiglitazone and LA-12 (S5 Fig). Inhibition of ERK pathway did not affect the rate of apoptosis induced by the combination of rosiglitazone and LA-12, as indicated by similar PARP cleavage (S5 Fig) and percentage of HCT116 wt cells with specific phosphatidyl serine externalization following the combined drug treatment in the absence or the presence of U0126 (data not shown).

### PPARγ is largely dispensable for the rosiglitazone-mediated enhancement of LA-12-induced apoptosis

**Fig 6:**
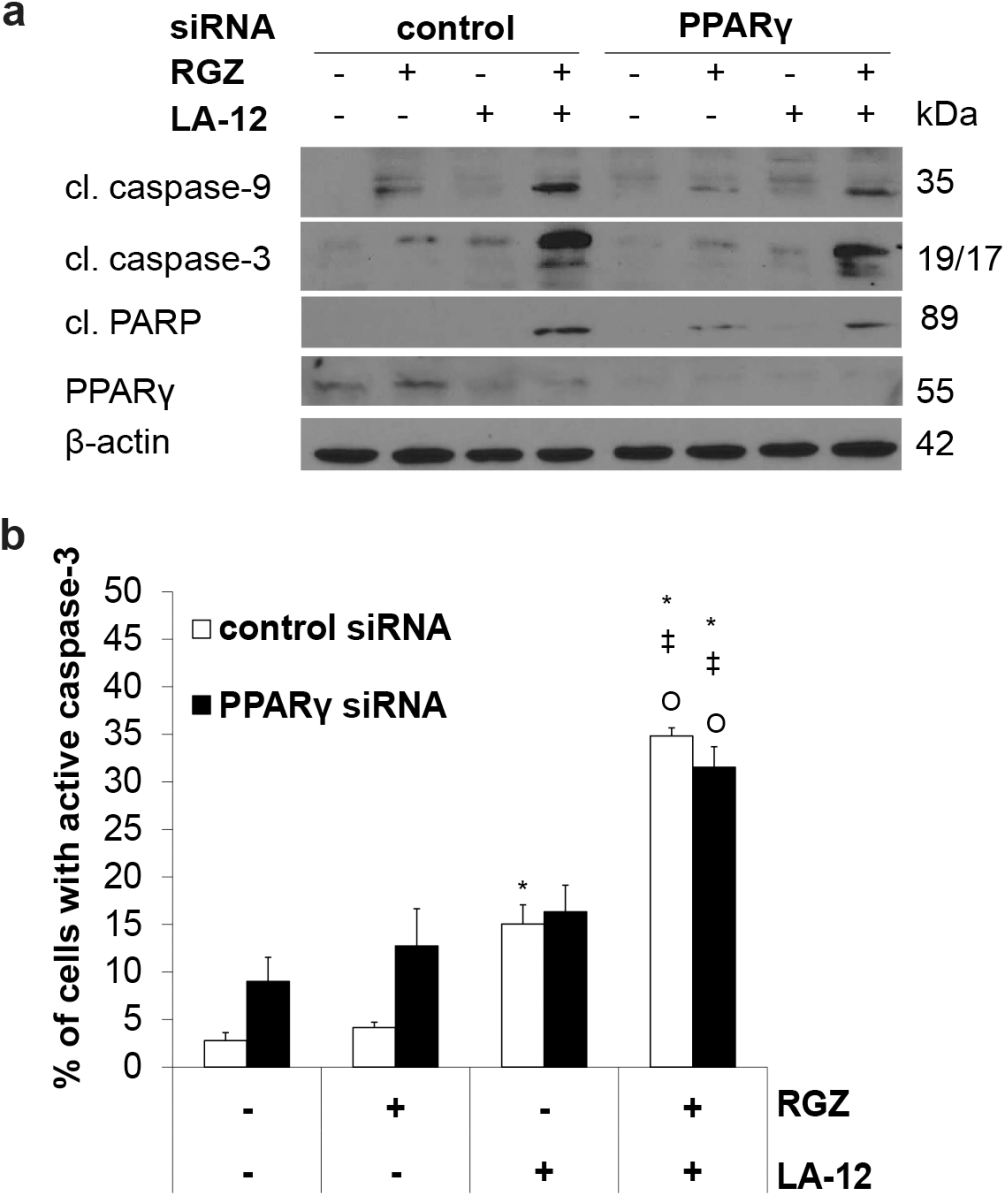
*A potential involvement of PPAR*γ *in apoptosis induced by combination of rosiglitazone and LA-12*. (a) Cleavage of caspase-9, caspase-3, PARP (Western blotting), and (b) caspase-3 activation (flow cytometry) in HCT116 wt cells transfected (24 h) with control or PPARγ siRNA, treated (24 h) with rosiglitazone (RGZ, 50 μM) and subsequently incubated (48 h) with LA-12 (0.75 μM). Results are means + S.E.M. of three independent experiments. Statistical significance: P < 0.05, * versus control, ‡ versus RGZ, Ο versus LA-12.

A specific silencing of PPARγ in HCT116 cells did not prevent apoptosis induced by rosiglitazone and LA-12 combination, as a similar cleavage of caspase-3, -9 and PARP (Fig 6A) and caspase-3 activation (Fig 6B) were apparent in PPARγ siRNA-treated HCT116 cells incubated with the drug combination, and their control siRNA-treated counterparts.

### The NCM460 cells derived from normal colon epithelium are resistant to the cytotoxic effects of the combination of rosiglitazone and LA-12

In contrast to HCT116, DLD-1 and RKO cancer cells, the same combination of rosiglitazone and LA-12 did not exert any apoptosis-inducing potential in NCM460 cells, as evidenced by the absence of PARP cleavage (S6A Fig) and caspase-3 activation (S6B Fig). A substantially different modulation of the NCM460 cell cycle progression was apparent following their treatment with rosiglitazone and LA-12 compared to the similarly treated HCT116 cell line. No significant changes in G2/M phase representation were detected, and the drug combination also did not significantly decrease the percentage of the NCM460 cells in the G1 phase (S6C Fig).

## Discussion

Although numerous studies investigated the cytotoxic/cytostatic response induced by rosiglitazone in various tumours [31], less is known about its ability to modulate the cancer cell sensitivity to the action of various chemotherapeutic drugs. Only several studies have reported on a cooperative anticancer effects of rosiglitazone and conventionally used platinum-based chemotherapeutic drugs such as cisplatin or carboplatin [13–15]. Moreover, the combined action of rosiglitazone and recently introduced platinum-based complexes such as LA-12 has been completely unexplored. Here we provide the first evidence that rosiglitazone significantly increased the cytotoxic effects of LA-12 in HCT116 human colon cancer cells via stimulation of caspase-dependent apoptosis, which was also associated with significant modulation of the cell cycle progression. For the combined treatments, a sub-toxic dose (50 μM) of rosiglitazone was used, as demonstrated by an absence of caspase activation, PARP cleavage or phosphatidyl serine externalization during the whole course of the HCT116 cell individual treatment with the drug. This is in agreement with Dai et al., who showed that similar concentrations of rosiglitazone did not significantly affect the above-mentioned apoptotic parameters in this cell line [32]. Importantly, the apoptosis sensitizing action of rosiglitazone was markedly apparent even when combined with a relatively low concentration of LA-12. Previously, we demonstrated an outstanding apoptosis-inducing or -sensitizing ability of LA-12 in colon cancer cells when used in significantly lower doses compared to some conventionally used platinum drugs, making it an attractive candidate for chemotherapy of colon tumours including those resistant to cisplatin or oxaliplatin [21, 24]. Thus, we presumed that the combination of favourable doses of rosiglitazone and LA-12 might reduce limiting toxicities to the healthy tissue. Our results showing the absence of the toxicity of the drug combination in NCM460 cells suggest that it could be safer for the cells derived from normal epithelium.

As an effective stimulation of colon cancer cell apoptosis triggered by the combination of rosiglitazone and LA-12 was associated with activation of mitochondrial pathway (cytochrome c release, drop of MMP, and caspase-9 activation), an involvement of these organelles was further examined in more detail using the cells lacking Bax protein. In this model, we newly showed an indispensable role of mitochondria in the cooperative apoptotic action of rosiglitazone and LA-12. In HCT116 cells treated with LA-12, an increase of the level of Noxa, Bim, Bax and Bak was detected, which is in agreement with our previously published results showing the LA-12-medited upregulation of several Bcl-2 family protein levels [24]. An additional increase in Bim and especially Noxa level was further observed in the cells treated with the drug combination, suggesting that these might contribute to the stimulation of mitochondrial apoptotic pathway. However, we observed that siRNA-mediated individual silencing of Noxa did not significantly affect the progress of HCT116 cell apoptosis induced by the drug combination (data not shown).

Our results further showed a profound downregulation of PTEN level associated with Akt activation in HCT116 cells treated with the combination of rosiglitazone and LA-12. Interestingly, we also observed that the PTEN deficiency rendered the cells more sensitive to apoptosis induction triggered by the drug combination, which was associated with higher activation of mitochondrial pathway. This finding was rather surprising as the positive impact of PTEN on activation of mitochondrial pathway has been mostly reported, closely associated with inhibition of Akt [33]. Moreover, we cannot exclude that the negative role of PTEN in regulation of apoptosis induced by the combination of rosiglitazone and LA-12 may also be mediated independently on Akt by other PTEN targets capable to modulate mitochondrial events important in the cooperative action of the two drugs.

We documented that the stimulation of apoptosis induced by the drug combination was accompanied by the significant changes in the cell cycle progression. Our previous work focused on the regulation of platinum drug-induced changes of the cell cycle showed oxaliplatin- and LA-12- induced increase of the percentage of HCT116 cells in G2/M phase [22]. However, in contrast to oxaliplatin, LA-12 allowed the cells to bypass the G2-phase arrest and enter M-phase, which was associated with its higher anticancer activity. In our present work, LA-12 also induced an increase in the number of G2/M-phase HCT116 PTEN+/+ cells, which was still apparent following its co-treatment with rosiglitazone. A further increase in percentage of HCT116 cells in G2/M phase induced by LA-12 or its combination with rosiglitazone was even more obvious in the absence of PTEN. In human A549 lung cancer cells, combination of rosiglitazone and carboplatin caused an increase of their number in G2/M phase compared to the individual drug treatments [14]. Pretreatment with rosiglitazone has also been shown to prolong radiation-induced G2/M arrest and growth inhibition in human colon cancer cells [9]. An interesting drop in cyclin B1 and survivin protein level following the incubation with LA-12 and rosiglitazone indicated that the PTEN+/+ and even more PTEN-/- cells affected by the drug combination may proceed faster to the late M phase, where the amount of these proteins usually tends to decline. Furthermore, a higher level of cyclin D1 protein in LA-12 and rosiglitazone-treated PTEN-/- cells compared to PTEN+/+ counterparts suggests that the cells affected by the drug combination in the absence of PTEN may occur more frequently in G1 phase. Loss of PTEN-induced Akt activation has been shown to overcome a DNA damage-induced G2/M checkpoint and facilitate further transition of colon cancer cells to G1 phase [34]. In addition, a faster transition from G2/M to G1 phase of the cell cycle was observed in PTEN null embryonic stem cells compared to their wt counterparts [34]. We suggest that for the PTEN-deficient cells treated with the combination of rosiglitazone and LA-12, it might be easier to pass through the G2/M phase of the cell cycle and eventually end up in G1 phase where undergo apoptosis, as confirmed by simultaneous flow cytometry analysis of the cell cycle progression and caspase-3 cleavage. The more pronounced decrease in p21 and p27 protein levels in the drug combination-treated PTEN-/- cells may reflect their more frequent dying during the progression via G1 phase of the cell cycle compared to the PTEN+/+ counterparts.

As p53 and Chk2 can act as important regulators of both the cell cycle progression and/or apoptosis, we examined their role in the cooperative action of the drugs. Using HCT116 cells deficient for p53 or Chk2, we showed that these two proteins are dispensable for cooperative proapoptotic effects of combination of LA-12 and rosiglitazone. Moreover, the dispensable role of p53 in the cytotoxic effects of the drug combination was further confirmed in DLD-1 cells with mutated p53. We and others previously reported that the cytotoxic effects of LA-12 on various types of cancer cells can be independent or only partially dependent on p53 status [19, 20, 22, 25, 35]. Here we extended our findings also for the combination of LA-12 with rosiglitazone, making it attractive candidate for effective elimination of colon cancer cells with non-functional p53.

We observed that rosiglitazone induced an early increase in nuclear translocation of PPARγ (confocal microscopy) and a total PPARγ level (Western blotting) in HCT116 cells (data not shown), followed by its marked decline in the later time points. However, as the siRNA-mediated downregulation of PPARγ failed to significantly inhibit the apoptotic effects induced by rosiglitazone and LA-12, we concluded that the drug combination-mediated cell death was mostly independent on PPARγ activation. A dispensable role of PPARγ in rosiglitazone-mediated sensitization of human renal cancer cells to TRAIL-induced apoptosis was also demonstrated using a dominant-negative mutant of PPARγ [11]. An accumulating evidence suggests that the growth-inhibitory and apoptotic effects of rosiglitazone may often be independent on PPARγ, as documented in non-small cell lung carcinoma and gastric cancer cells [36, 37]. Similarly as for rosiglitazone, PPARγ-independent anticancer action of other synthetic PPARγ ligands such as troglitazone or ciglitazone has been reported in colon, prostate or bladder cancer cells [38–40], but the underlying mechanisms and the general frequency of these effects remain to be elucidated. Some studies also reported on the ability of PPARγ ligands to activate ERK cascade, often via PPARγ-independent signalling [41]. Although an apparent increase in ERK1/2 phosphorylation was observed by us in HCT116 cells treated with rosiglitazone and LA-12, the functional relevance of ERK pathway in the apoptotic action of the drug combination has been excluded.

We showed that pretreatment of HCT116 colon cancer cells with rosiglitazone resulted in profound selective enhancement of LA-12-induced caspase-dependent apoptosis associated with significant activation of mitochondrial pathway. The apoptosis induced by the combination of rosiglitazone and LA-12 occurs preferentially in the G1 cell cycle phase, independently on p53 protein, and was accompanied with significant changes of selected Bcl-2 family protein levels, activation of specific kinases, and changes in the cell cycle progression. Interestingly, further stimulation of cooperative synergic apoptotic effects of rosiglitazone and LA-12 was demonstrated in the absence of PTEN, which makes it an important factor affecting the drug combination-induced cytotoxicity. In addition, the rosiglitazone-mediated sensitization to LA-12-induced apoptosis was demonstrated in several human colon adenocarcinoma cell lines, further underscoring the general relevance of our findings. Our results suggest that combined treatment with rosiglitazone and LA-12 might be promising therapeutic strategy in colon-derived tumours regardless of their p53 status, and further more favourable in those defective in PTEN function. Understanding the molecular mechanisms involved in the action of the new drug combination could help us to contribute to development of better strategies to combat this type of cancer.

## Supporting Information

S1 Fig Percentage of apoptotic (annexin V positive/propidium iodide negative, flow cytometry) HCT116 wt cells pretreated (24 h) with rosiglitazone (RGZ, 50 μM) and subsequently treated (48 h) with LA-12 (0.75 μM), in the absence (DMSO) or presence of z-VAD-fmk (10 μM). Results are means + S.E.M of three independent experiments. Statistical significance: P < 0.05, * versus control, ‡ versus RGZ, Ο versus LA-12, and Δ for with/without z-VAD-fmk.
(TIF)

S2 Fig The level of PTEN, phosphorylated and total level of Akt (Western blotting) in HCT116 wt cells pretreated (24 h) with rosiglitazone (RGZ, 50 μM), and subsequently treated (48 h) with LA-12 (0.75 μM). Results are representatives of at least three independent experiments.
(TIF)

S3 Fig The level of cyclin D1, p21, p27, cyclin B1 and survivin (Western blotting) in HCT116 wt or PTEN-/- cells pretreated (24 h) with rosiglitazone (RGZ, 50 μM), and subsequently treated (48 h) with LA-12 (0.75 μM). Results are representatives of at least three independent experiments.
(TIF)

S4 Fig The relative level of CCDN1 (cyclin D1), CDKN1A (p21), CDKN1B (p27), CCNB1 (cyclin B1) and BIRC5 (survivin) mRNA in HCT116 PTEN +/+ or -/- cells pretreated (24 h) with rosiglitazone (RGZ, 50 μM), and subsequently treated (48 h) with LA-12 (0.75 μM), detected by quantitative real-time polymerase chain reaction, appropriate control = 1. Results are means + S.E.M. or representatives of three independent experiments. Statistical significance: P < 0.05, * versus control, ‡ versus RGZ, Ο versus LA-12, and Δ for PTEN+/+ versus PTEN-/- cells.
(TIF)

S5 Fig Cleavage of PARP, phosphorylated and total ERK1/2 level (Western blotting) in HCT116 wt cells pretreated (24 h) with rosiglitazone (RGZ, 50 μM) and subsequently treated (48 h) with LA-12 (0.75 μM), in the absence (DMSO) or presence of U0126 (10 μM). Results are representatives of at least three independent experiments.
(TIF)

S6 Fig (a) PARP cleavage (Western blotting) in HCT116 wt and NCM460 cells pretreated (24 h) with rosiglitazone (RGZ, 50 μM) and subsequently treated (48 h) with LA-12 (0.75 μM). (b) Caspase-3 activity (flow cytometry) in NCM460 cells treated as in a). Results are means + S.E.M. of three independent experiments. Positive control represents the cells treated (72 h) with DHA (50 μM). (c) The percentage of NCM460 cells in individual cell cycle phases (flow cytometry) following their pretreatment (24 h) with rosiglitazone (RGZ, 50 μM), and subsequent treatment (48 h) with LA-12 (0.75 μM). Results are means + S.E.M of three independent experiments. Statistical significance: P < 0.05, * versus control, ‡ versus RGZ or Ο versus LA-12.
(TIF)

S7 Fig Original blots with markers for results presented in Fig 1. (TIF)

S8 Fig Original blots with markers for results presented in Fig 2. (TIF)

S9 Fig Original blots with markers for results presented in Fig 4. (TIF)

S10 Fig Original blots with markers for results presented in Fig 5. (TIF)

S11 Fig Original blots with markers for results presented in Fig 6. (TIF)

S12 Fig Original blots with markers for results presented in S2 Fig. (TIF)

S13 Fig Original blots with markers for results presented in S3 Fig. (TIF)

S14 Fig Original blots with markers for results presented in S5 Fig. (TIF)

S15 Fig Original blots with markers for results presented in S6 Fig. (TIF)

## References

[1] Grygiel-Gorniak B. Peroxisome proliferator-activated receptors and their ligands: nutritional and clinical implications -a review. Nutr J. 2014;13:17. Epub 2014/02/15. doi: 1475-2891-13-17 [pii] 10.1186/1475-2891-13-17 . 24524207 PMC3943808

[2] Siersbaek R, Nielsen R, Mandrup S. PPARγin adipocyte differentiation and metabolism- novel insights from genome-wide studies. FEBS Lett. 2010;584(15):3242–9. Epub 2010/06/15. doi: S0014-5793(10)00492-8 [pii] 10.1016/j.febslet.2010.06.010 . 20542036

[3] Brockman JA, Gupta RA, Dubois RN. Activation of PPARγ leads to inhibition of anchorage-independent growth of human colorectal cancer cells. Gastroenterology. 1998;115(5):1049–55. Epub 1998/10/31. doi: S0016508598004521 [pii]. . 9797355 10.1016/s0016-5085(98)70072-1

[4] Fajas L, Egler V, Reiter R, Miard S, Lefebvre AM, Auwerx J. PPARγ controls cell proliferation and apoptosis in an RB-dependent manner. Oncogene. 2003;22(27):4186–93. Epub 2003/07/02. 10.1038/sj.onc.1206530 1206530 [pii]. . 12833141

[5] Lefebvre M, Paulweber B, Fajas L, Woods J, McCrary C, Colombel JF, et al Peroxisome proliferator-activated receptor γ is induced during differentiation of colon epithelium cells. J Endocrinol. 1999;162(3):331–40. Epub 1999/09/01. 10.1677/joe.0.1620331 . 10467224

[6] Yang WL, Frucht H. Activation of the PPAR pathway induces apoptosis and COX-2 inhibition in HT-29 human colon cancer cells. Carcinogenesis. 2001;22(9):1379–83. Epub 2001/09/05. 10.1093/carcin/22.9.1379 . 11532858

[7] Sarraf P, Mueller E, Jones D, King FJ, DeAngelo DJ, Partridge JB, et al Differentiation and reversal of malignant changes in colon cancer through PPARγ. Nat Med. 1998;4(9):1046–52. Epub 1998/09/12. 10.1038/2030 . 9734398

[8] Ikezoe T, Miller CW, Kawano S, Heaney A, Williamson EA, Hisatake J, et al Mutational analysis of the peroxisome proliferator-activated receptor γ gene in human malignancies. Cancer Res. 2001;61(13):5307–10. Epub 2001/06/30. . 11431375

[9] Chiu SJ, Hsaio CH, Tseng HH, Su YH, Shih WL, Lee JW, et al Rosiglitazone enhances the radiosensitivity of p53-mutant HT-29 human colorectal cancer cells. Biochem Biophys Res Commun. 2010;394(3):774–9. Epub 2010/03/17. doi: S0006-291X(10)00519-X [pii] 10.1016/j.bbrc.2010.03.068 . 20227390

[10] Zhang YQ, Tang XQ, Sun L, Dong L, Qin Y, Liu HQ, et al Rosiglitazone enhances fluorouracil-induced apoptosis of HT-29 cells by activating peroxisome proliferator-activated receptor γ. World J Gastroenterol. 2007;13(10):1534–40. Epub 2007/04/28. 10.3748/wjg.v13.i10.1534 . 17461445 PMC4146895

[11] Kim YH, Jung EM, Lee TJ, Kim SH, Choi YH, Park JW, et al Rosiglitazone promotes tumor necrosis factor-related apoptosis-inducing ligand-induced apoptosis by reactive oxygen species-mediated up-regulation of death receptor 5 and down-regulation of c-FLIP. Free Radic Biol Med. 2008;44(6):1055–68. Epub 2008/01/01. doi: S0891-5849(07)00797-6 [pii] 10.1016/j.freeradbiomed.2007.12.001 . 18164688

[12] Miao R, Xu T, Liu L, Wang M, Jiang Y, Li J, et al Rosiglitazone and retinoic acid inhibit proliferation and induce apoptosis in the HCT-15 human colorectal cancer cell line. Exp Ther Med. 2011;2(3):413–7. Epub 2011/05/01. 10.3892/etm.2011.227 etm-02-03-0413 [pii]. . 22977519 PMC3440746

[13] Girnun GD, Naseri E, Vafai SB, Qu L, Szwaya JD, Bronson R, et al Synergy between PPARγ ligands and platinum-based drugs in cancer. Cancer Cell. 2007;11(5):395–406. Epub 2007/05/08. doi: S1535-6108(07)00066-9 [pii] 10.1016/j.ccr.2007.02.025 . 17482130 PMC2564847

[14] Girnun GD, Chen L, Silvaggi J, Drapkin R, Chirieac LR, Padera RF, et al Regression of drug-resistant lung cancer by the combination of rosiglitazone and carboplatin. Clin Cancer Res. 2008;14(20):6478–86. Epub 2008/10/18. doi: 14/20/6478 [pii] 10.1158/1078-0432.CCR-08-1128 . 18927287 PMC2696122

[15] Tikoo K, Kumar P, Gupta J. Rosiglitazone synergizes anticancer activity of cisplatin and reduces its nephrotoxicity in 7, 12-dimethyl benz{a}anthracene (DMBA) induced breast cancer rats. BMC Cancer. 2009;9:107. Epub 2009/04/10. doi: 1471-2407-9-107 [pii] 10.1186/1471-2407-9-107 . 19356226 PMC2676298

[16] Zak F, Turanek J, Kroutil A, Sova P, Mistr A, Poulova A, et al Platinum(IV) complex with adamantylamine as nonleaving amine group: synthesis, characterization, and in vitro antitumor activity against a panel of cisplatin-resistant cancer cell lines. J Med Chem. 2004;47(3):761–3. Epub 2004/01/23. 10.1021/jm030858+ . 14736257

[17] Kozubik A, Vaculova A, Soucek K, Vondracek J, Turanek J, Hofmanova J. Novel anticancer platinum(IV) complexes with adamantylamine: their efficiency and innovative chemotherapy strategies modifying lipid metabolism. Met Based Drugs. 2008;2008:417897 Epub 2008/04/17. 10.1155/2008/417897 . 18414587 PMC2291354

[18] Kozubik A, Horvath V, Svihalkova-Sindlerova L, Soucek K, Hofmanova J, Sova P, et al High effectiveness of platinum(IV) complex with adamantylamine in overcoming resistance to cisplatin and suppressing proliferation of ovarian cancer cells in vitro. Biochem Pharmacol. 2005;69(3):373–83. Epub 2005/01/18. doi: S0006-2952(04)00646-X [pii] 10.1016/j.bcp.2004.09.005 . 15652229

[19] Horvath V, Blanarova O, Svihalkova-Sindlerova L, Soucek K, Hofmanova J, Sova P, et al Platinum(IV) complex with adamantylamine overcomes intrinsic resistance to cisplatin in ovarian cancer cells. Gynecol Oncol. 2006;102(1):32–40. Epub 2005/12/21. doi: S0090-8258(05)01006-1 [pii] 10.1016/j.ygyno.2005.11.016 . 16364413

[20] Roubalova E, Kvardova V, Hrstka R, Borilova S, Michalova E, Dubska L, et al The effect of cellular environment and p53 status on the mode of action of the platinum derivative LA-12. Invest New Drugs. 2010;28(4):445–53. Epub 2009/06/06. 10.1007/s10637-009-9270-4 . 19499188

[21] Svihalkova-Sindlerova L, Foltinova V, Vaculova A, Horvath V, Soucek K, Sova P, et al LA-12 overcomes confluence-dependent resistance of HT-29 colon cancer cells to Pt (II) compounds. Anticancer Res. 2010;30(4):1183–8. Epub 2010/06/10. doi: 30/4/1183 [pii]. . 20530425

[22] Vondalova Blanarova O, Jelinkova I, Hyrslova Vaculova A, Sova P, Hofmanova J, Kozubik A. Higher anti-tumour efficacy of platinum(IV) complex LA-12 is associated with its ability to bypass M-phase entry block induced in oxaliplatin-treated human colon cancer cells. Cell Prolif. 2013;46(6):665–76. Epub 2013/10/15. 10.1111/cpr.12061 . 24118195 PMC6496914

[23] Vondalova Blanarova O, Jelinkova I, Szoor A, Skender B, Soucek K, Horvath V, et al Cisplatin and a potent platinum(IV) complex-mediated enhancement of TRAIL-induced cancer cells killing is associated with modulation of upstream events in the extrinsic apoptotic pathway. Carcinogenesis. 2011;32(1):42–51. Epub 2010/11/03. doi: bgq220 [pii] 10.1093/carcin/bgq220 . 21037225

[24] Jelinkova I, Safarikova B, Vondalova Blanarova O, Skender B, Hofmanova J, Sova P, et al Platinum(IV) complex LA-12 exerts higher ability than cisplatin to enhance TRAIL-induced cancer cell apoptosis via stimulation of mitochondrial pathway. Biochem Pharmacol. 2014;92(3):415–24. Epub 2014/10/07. doi: S0006-2952(14)00557-7 [pii] 10.1016/j.bcp.2014.09.013 . 25285768

[25] Kvardova V, Hrstka R, Walerych D, Muller P, Matoulkova E, Hruskova V, et al The new platinum(IV) derivative LA-12 shows stronger inhibitory effect on Hsp90 function compared to cisplatin. Mol Cancer. 2010;9:147. Epub 2010/06/17. doi: 1476-4598-9-147 [pii] 10.1186/1476-4598-9-147 . 20550649 PMC2893458

[26] Sova P, Mistr A, Kroutil A, Zak F, Pouckova P, Zadinova M. Comparative anti-tumor efficacy of two orally administered platinum(IV) drugs in nude mice bearing human tumor xenografts. Anticancer Drugs. 2006;17(2):201–6. Epub 2006/01/24. doi: 00001813-200602000-00012 [pii]. . 16428939 10.1097/00001813-200602000-00012

[27] Bunz F, Dutriaux A, Lengauer C, Waldman T, Zhou S, Brown JP, et al Requirement for p53 and p21 to sustain G2 arrest after DNA damage. Science. 1998;282(5393):1497–501. Epub 1998/11/20. . 9822382 10.1126/science.282.5393.1497

[28] Lee C, Kim JS, Waldman T. PTEN gene targeting reveals a radiation-induced size checkpoint in human cancer cells. Cancer Res. 2004;64(19):6906–14. Epub 2004/10/07. doi: 64/19/6906 [pii] 10.1158/0008-5472.CAN-04-1767 . 15466180 PMC4384184

[29] Moyer MP, Manzano LA, Merriman RL, Stauffer JS, Tanzer LR. NCM460, a normal human colon mucosal epithelial cell line. In Vitro Cell Dev Biol Anim. 1996;32(6):315–7. Epub 1996/06/01. . 8842743 10.1007/BF02722955

[30] Vaculova A, Hofmanova J, Soucek K, Andera L, Kozubik A. Ethanol acts as a potent agent sensitizing colon cancer cells to the TRAIL-induced apoptosis. FEBS Lett. 2004;577(1–2):309–13. Epub 2004/11/06. doi: S0014579304012426 [pii] 10.1016/j.febslet.2004.10.013 . 15527805

[31] Elrod HA, Sun SY. PPAR and apoptosis in cancer. PPAR Res. 2008;2008:704165 Epub 2008/07/11. 10.1155/2008/704165 . 18615184 PMC2442903

[32] Dai Y, Qiao L, Chan KW, Zou B, Ma J, Lan HY, et al Loss of XIAP sensitizes rosiglitazone-induced growth inhibition of colon cancer in vivo. Int J Cancer. 2008;122(12):2858–63. Epub 2008/03/21. 10.1002/ijc.23443 . 18351648

[33] Zhu Y, Hoell P, Ahlemeyer B, Krieglstein J. PTEN: a crucial mediator of mitochondria-dependent apoptosis. Apoptosis. 2006;11(2):197–207. Epub 2006/02/28. 10.1007/s10495-006-3714-5 . 16502258

[34] Kandel ES, Skeen J, Majewski N, Di Cristofano A, Pandolfi PP, Feliciano CS, et al Activation of Akt/protein kinase B overcomes a G(2)/M cell cycle checkpoint induced by DNA damage. Mol Cell Biol. 2002;22(22):7831–41. Epub 2002/10/23. 12391152 10.1128/MCB.22.22.7831-7841.2002 PMC134727

[35] Hrstka R, Powell DJ, Kvardova V, Roubalova E, Bourougaa K, Candeias MM, et al The novel platinum(IV) complex LA-12 induces p53 and p53/47 responses that differ from the related drug, cisplatin. Anticancer Drugs. 2008;19(4):369–79. Epub 2008/05/06. 10.1097/CAD.0b013e3282f7f50000001813-200804000-00005 [pii]. . 18454047

[36] Han S, Roman J. Rosiglitazone suppresses human lung carcinoma cell growth through PPARγ-dependent and PPAR-independent signal pathways. Mol Cancer Ther. 2006;5(2):430–7. Epub 2006/03/01. doi: 5/2/430 [pii] 10.1158/1535-7163.MCT-05-0347 . 16505118

[37] He Q, Pang R, Song X, Chen J, Chen H, Chen B, et al Rosiglitazone suppresses the growth and invasiveness of SGC-7901 gastric cancer cells and angiogenesis in vitro via PPARγ dependent and independent mechanisms. PPAR Res. 2008;2008:649808 Epub 2008/09/24. 10.1155/2008/649808 . 18810275 PMC2542845

[38] Qiao L, Dai Y, Gu Q, Chan KW, Ma J, Lan HY, et al Loss of XIAP sensitizes colon cancer cells to PPARγ independent antitumor effects of troglitazone and 15-PGJ2. Cancer Lett. 2008;268(2):260–71. Epub 2008/05/15. doi: S0304-3835(08)00279-6 [pii] 10.1016/j.canlet.2008.04.003 . 18477501

[39] Shiau CW, Yang CC, Kulp SK, Chen KF, Chen CS, Huang JW. Thiazolidenediones mediate apoptosis in prostate cancer cells in part through inhibition of Bcl-xL/Bcl-2 functions independently of PPARγ. Cancer Res. 2005;65(4):1561–9. Epub 2005/03/01. doi: 65/4/1561 [pii] 10.1158/0008-5472.CAN-04-1677 . 15735046

[40] Chaffer CL, Thomas DM, Thompson EW, Williams ED. PPARγ-independent induction of growth arrest and apoptosis in prostate and bladder carcinoma. BMC Cancer. 2006;6:53. Epub 2006/03/08. doi: 1471-2407-6-53 [pii] 10.1186/1471-2407-6-53 . 16519808 PMC1450298

[41] Burgermeister E, Seger R. PPARγ and MEK Interactions in Cancer. PPAR Res. 2008;2008:309469 Epub 2008/07/04. 10.1155/2008/309469 . 18596912 PMC2440494

