# Supplementary figures and images for: Loss of PTEN Facilitates Rosiglitazone-Mediated Enhancement of Platinum(IV) Complex LA-12-Induced Apoptosis in Colon Cancer Cells
Source: PLoS One. 2015 Oct 22;10(10):e0141020. doi: 10.1371/journal.pone.0141020 (PMC4619604; doi:10.1371/journal.pone.0141020)

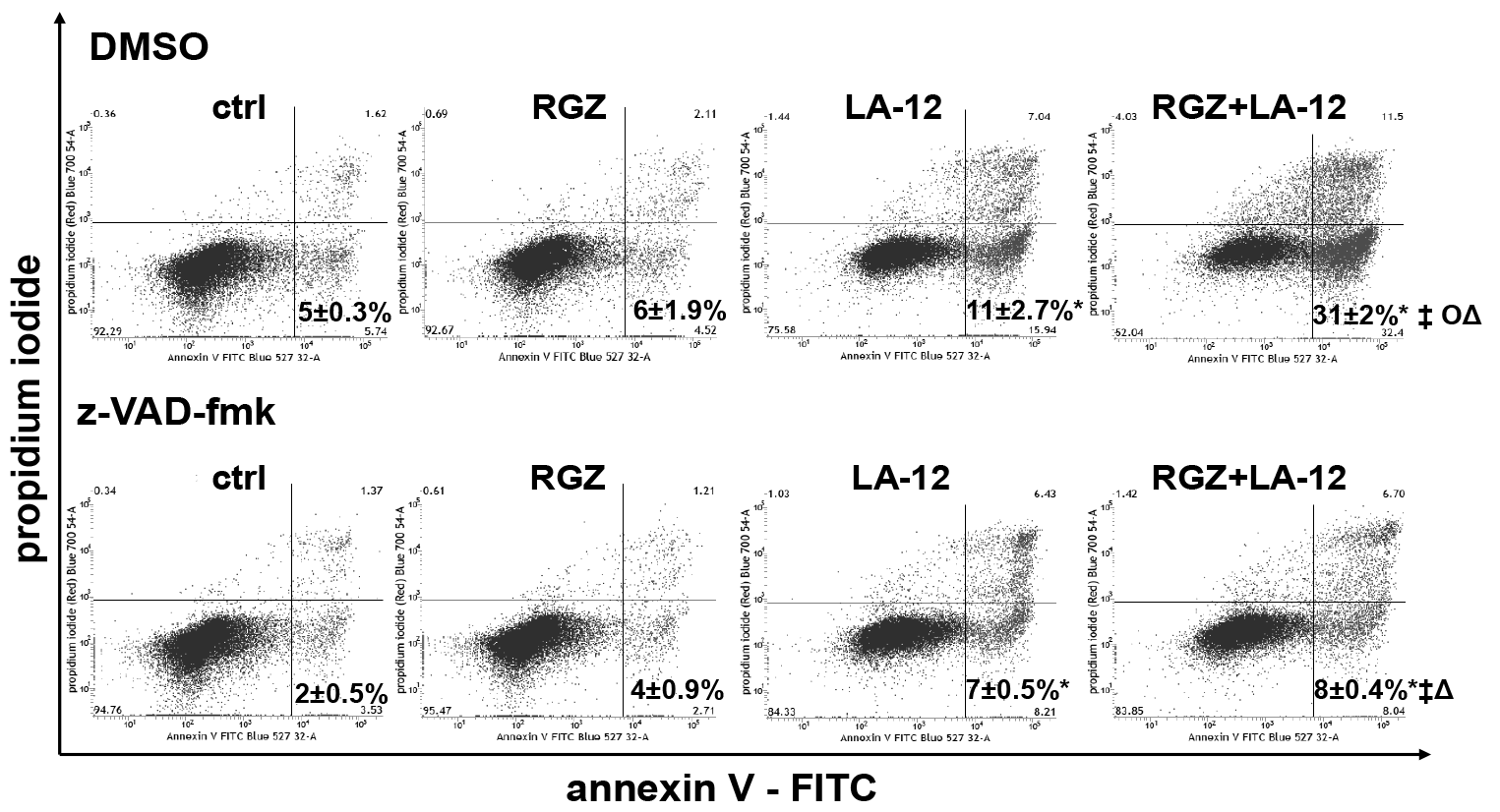

Supplement: S1 Fig — Results are means + S.E.M of three independent experiments. Statistical significance: P < 0.05, * versus control, ‡ versus RGZ, Ο versus LA-12, and Δ for with/without z-VAD-fmk. (TIF) [file pone.0141020.s001.tif]

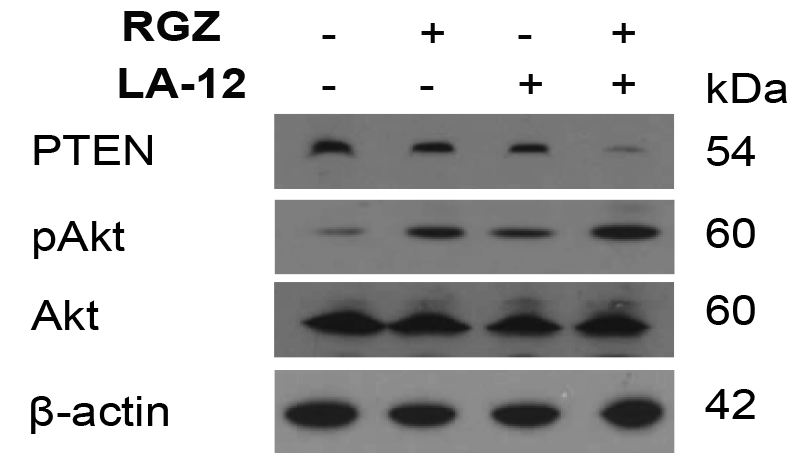

Supplement: S2 Fig — Results are representatives of at least three independent experiments. (TIF) [file pone.0141020.s002.tif]

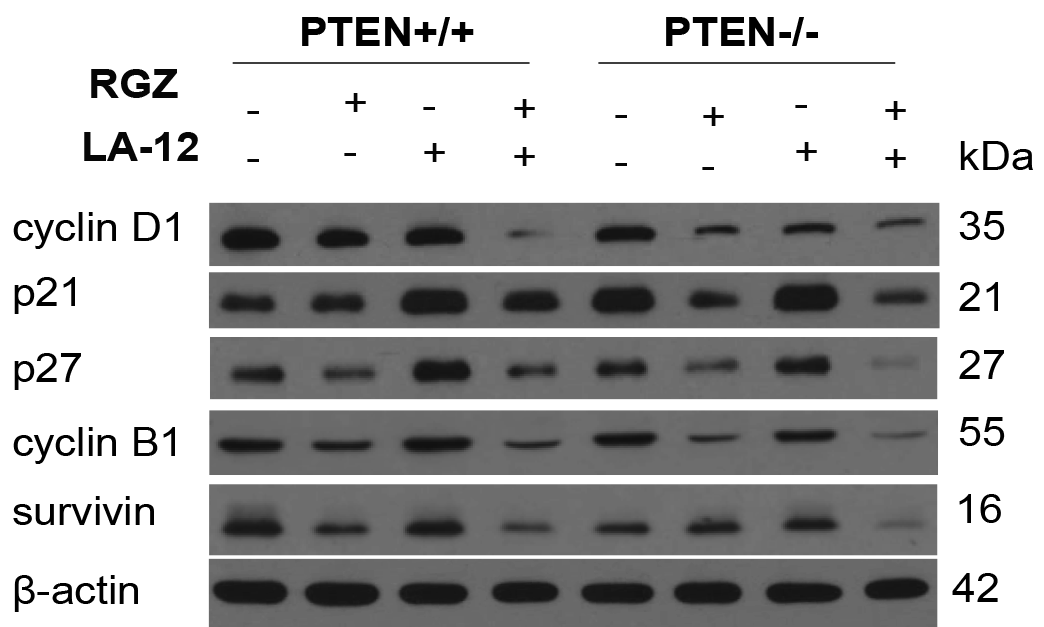

Supplement: S3 Fig — Results are representatives of at least three independent experiments. (TIF) [file pone.0141020.s003.tif]

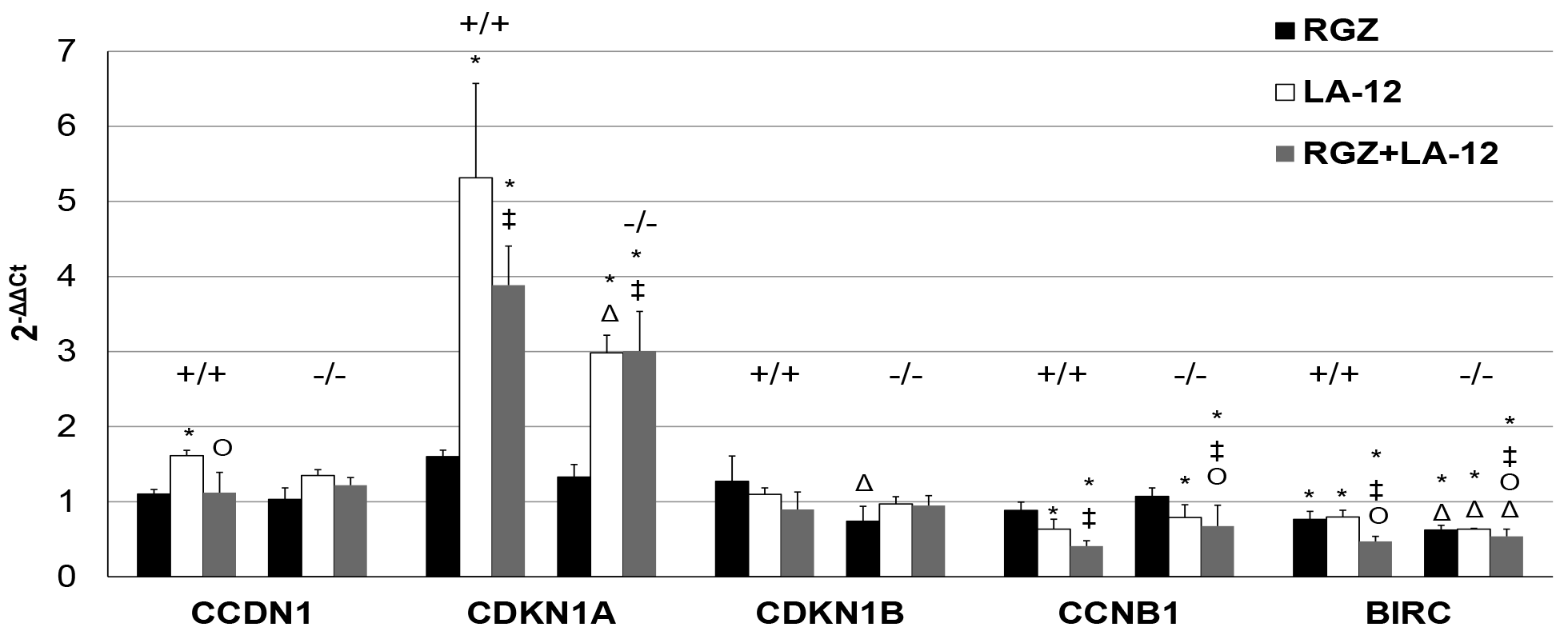

Supplement: S4 Fig — Results are means + S.E.M. or representatives of three independent experiments. Statistical significance: P < 0.05, * versus control, ‡ versus RGZ, Ο versus LA-12, and Δ for PTEN+/+ versus PTEN-/- cells. (TIF) [file pone.0141020.s004.tif]

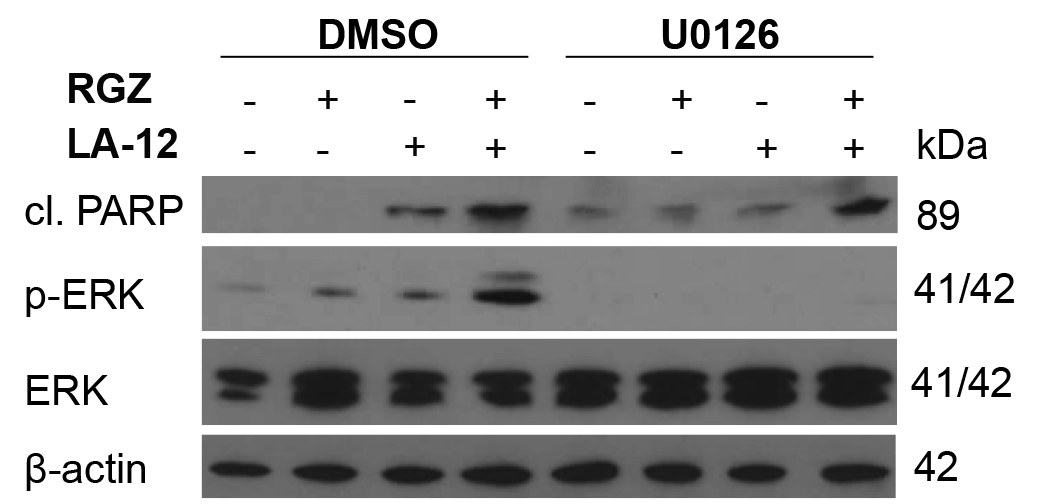

Supplement: S5 Fig — Results are representatives of at least three independent experiments. (TIF) [file pone.0141020.s005.tif]

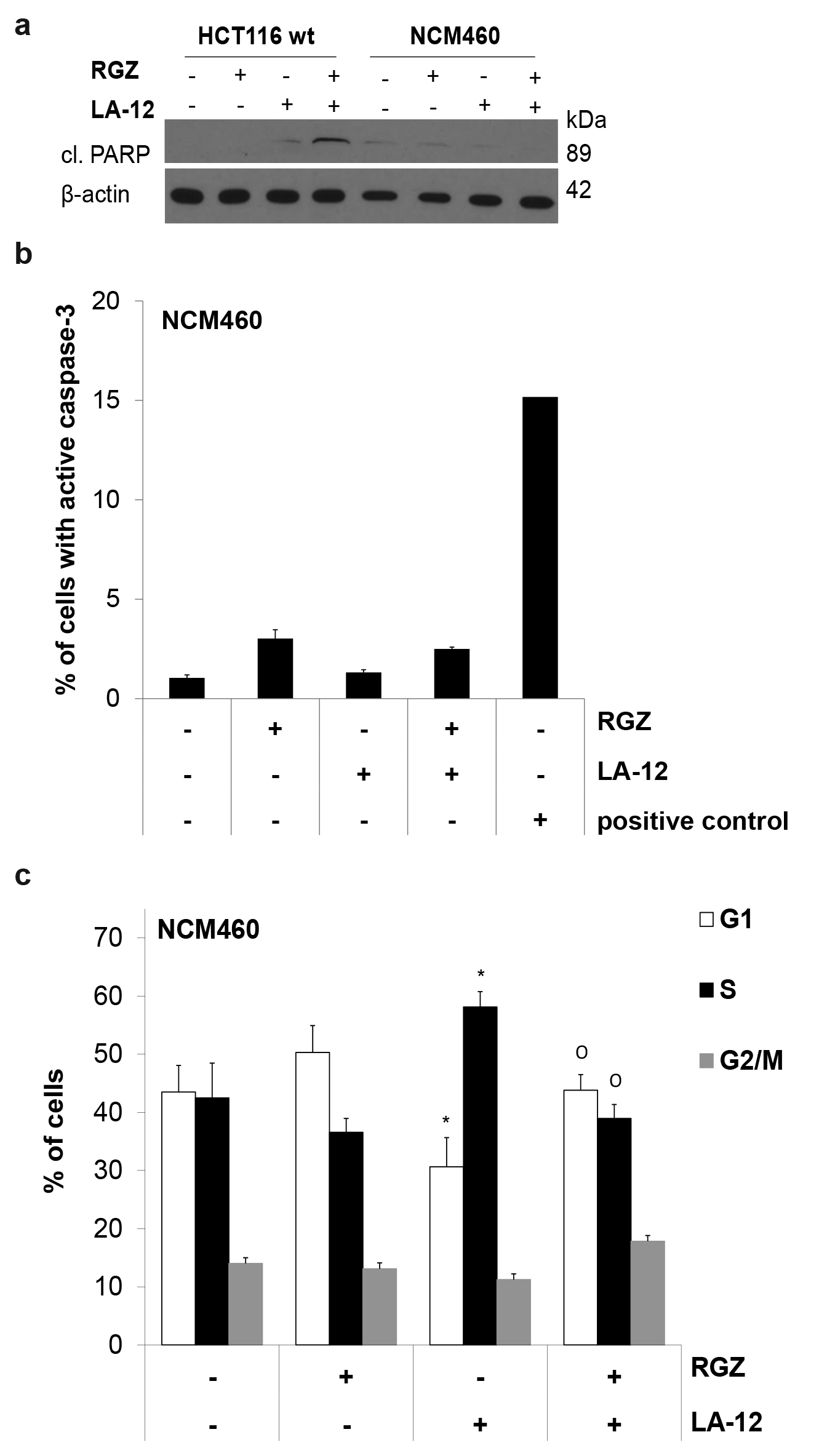

Supplement: S6 Fig — Statistical significance: P < 0.05, * versus control, ‡ versus RGZ or Ο versus LA-12. (TIF) [file pone.0141020.s006.tif]

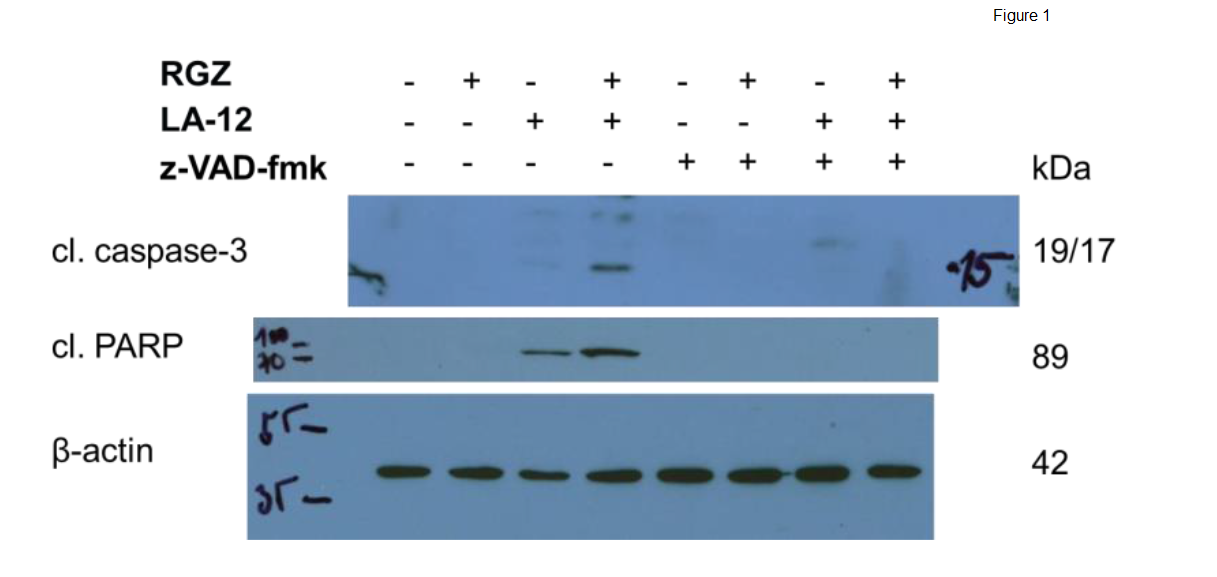

Supplement: S7 Fig — (TIF) [file pone.0141020.s007.tif]

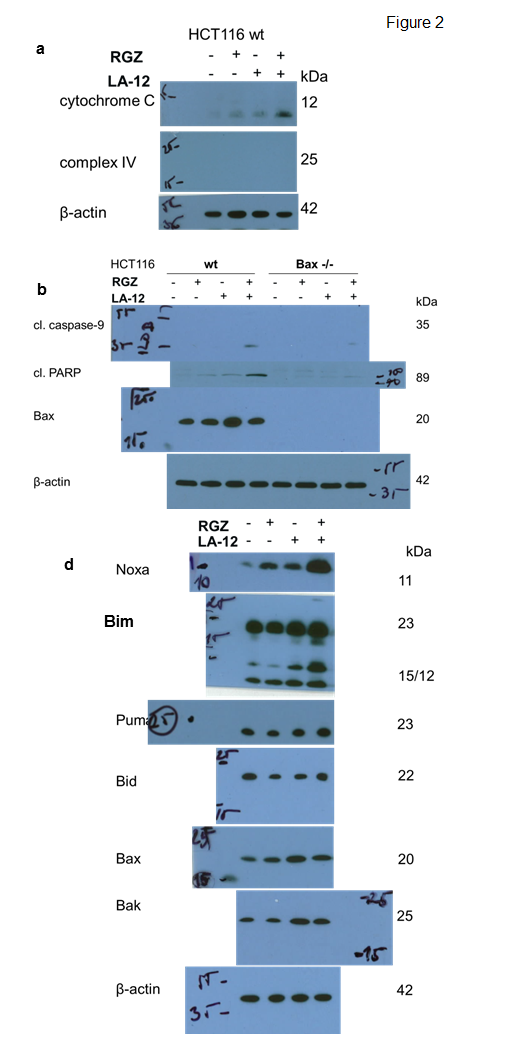

Supplement: S8 Fig — (TIF) [file pone.0141020.s008.tif]

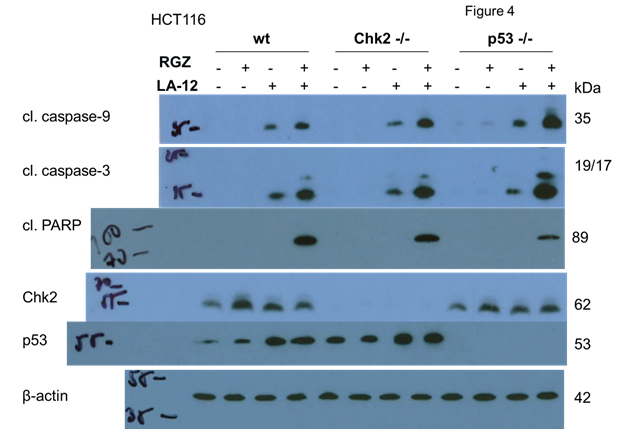

Supplement: S9 Fig — (TIF) [file pone.0141020.s009.tif]

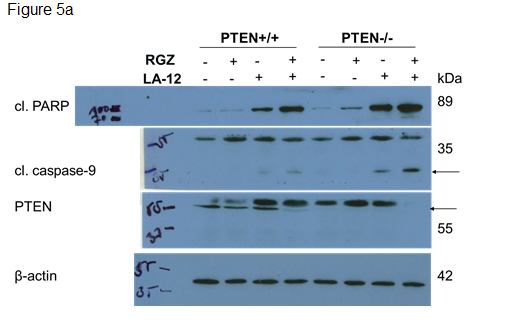

Supplement: S10 Fig — (TIF) [file pone.0141020.s010.tif]

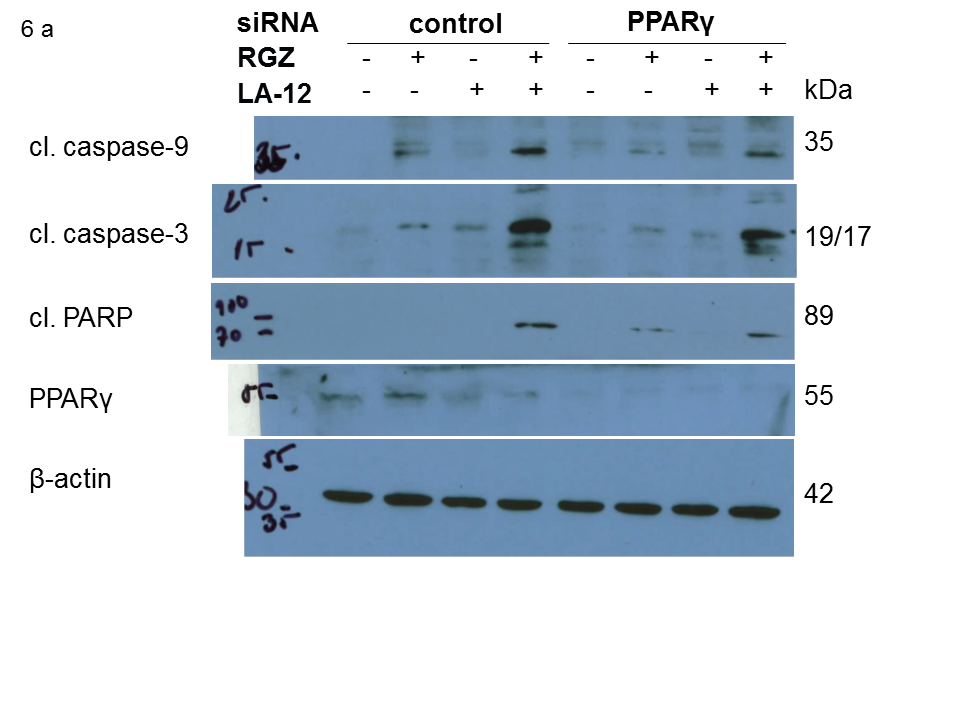

Supplement: S11 Fig — (TIF) [file pone.0141020.s011.tif]

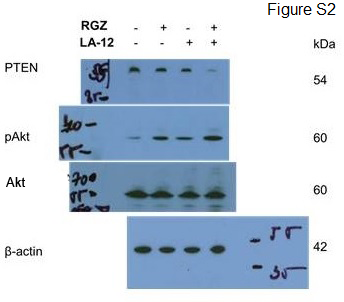

Supplement: S12 Fig — (TIF) [file pone.0141020.s012.tif]

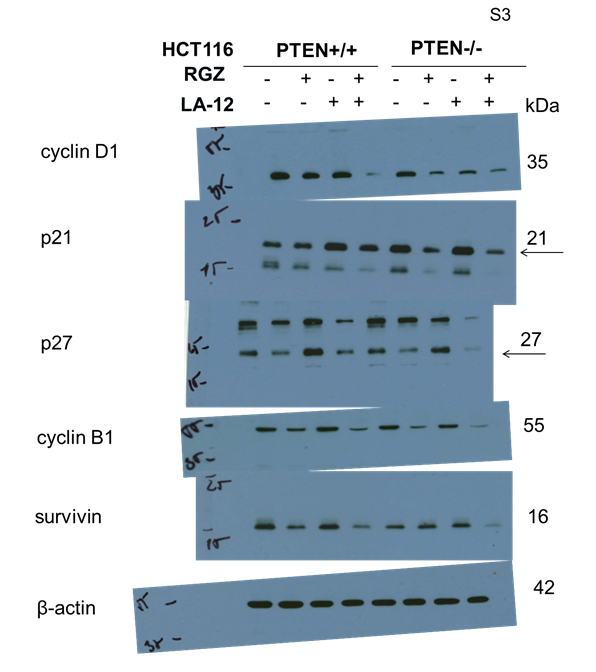

Supplement: S13 Fig — (TIF) [file pone.0141020.s013.tif]

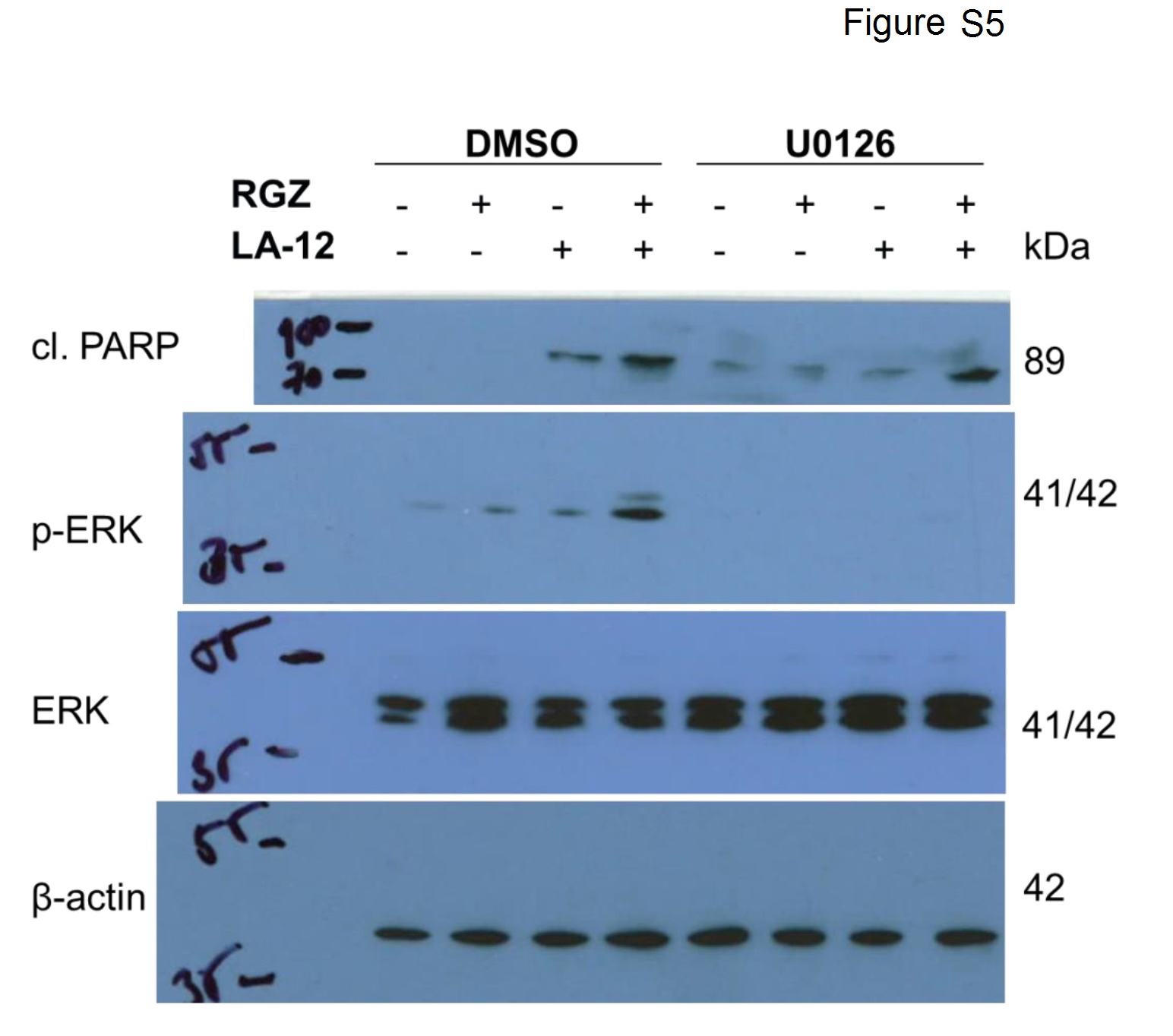

Supplement: S14 Fig — (TIF) [file pone.0141020.s014.tif]

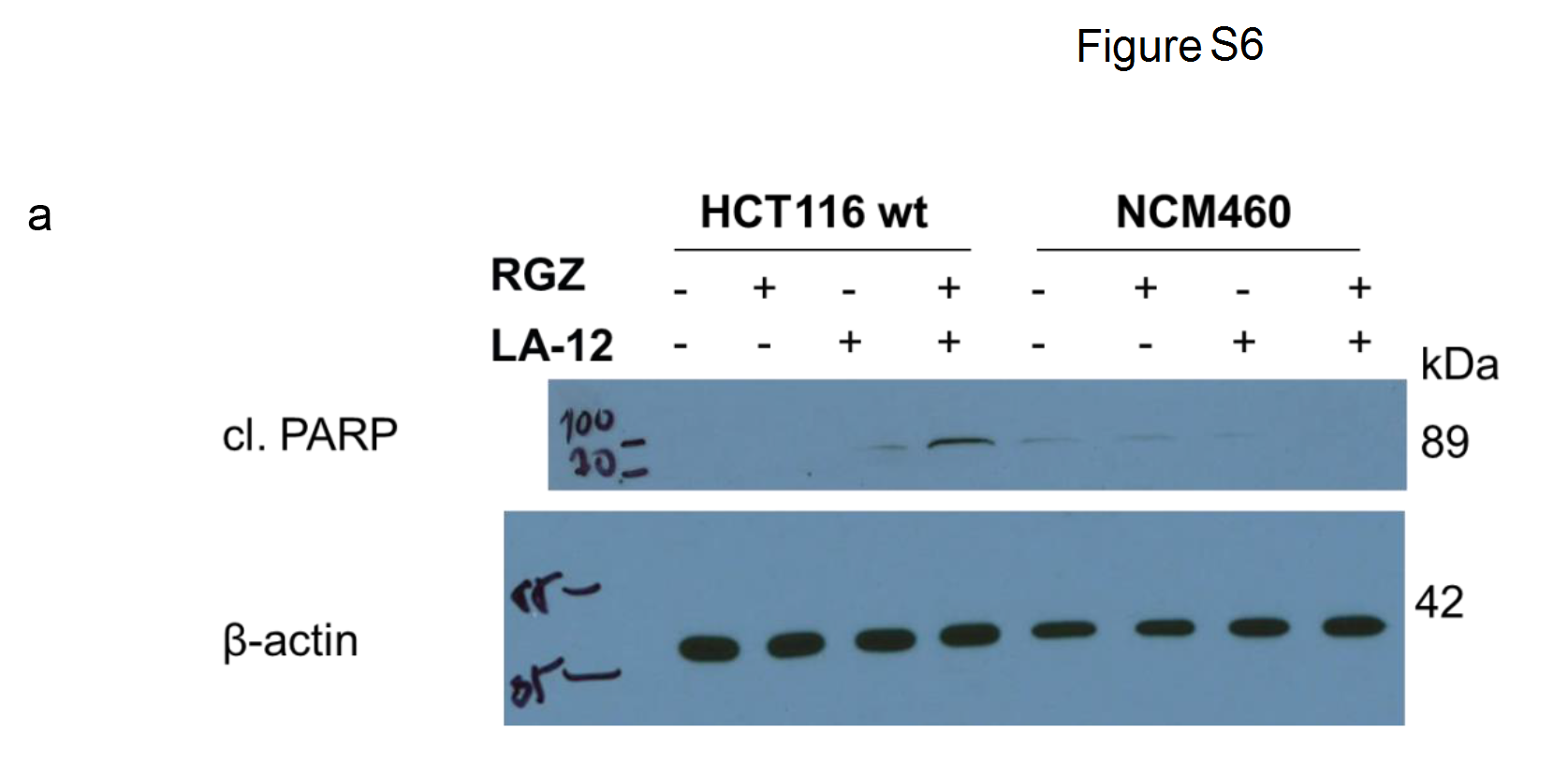

Supplement: S15 Fig — (TIF) [file pone.0141020.s015.tif]
